# Bridging cultures: the role of school’s cultural diversity climate and cultural sensitivity in immigrant parents’ school involvement

**DOI:** 10.3389/fpsyg.2025.1561863

**Published:** 2025-03-27

**Authors:** María José Mera-Lemp, José J. Pizarro, Florencia Guglielmetti-Serrano

**Affiliations:** ^1^Escuela de Ciencias Jurídicas y Sociales, Universidad Viña del Mar, Viña del Mar, Chile; ^2^Department of Social Psychology, University of the Basque Country UPV/EHU, Donostia, Spain; ^3^Escuela de Psicología, Universidad Católica del Norte, Antofagasta, Chile; ^4^Escuela de Psicología, Universidad Gabriela Mistral, Santiago, Chile

**Keywords:** immigrant parents, parental school involvement, cultural diversity climate, cultural sensitivity, intercultural education

## Abstract

Parents’ involvement in children’s education has been identified as a significant predictor of students’ school achievement and psychological outcomes. In the case of immigrant parents, research has suggested that low educational levels, poor incomes, and pertaining to minority cultural groups negatively affect their participation in both school and home academic activities. Nevertheless, little is known about the influence of schools’ approaches to cultural diversity and parents’ intercultural competencies on their commitment to children’s schooling processes. This study aims to examine the relationships between cultural diversity climate at school, cultural sensitivity, and school involvement among 751 Venezuelan and Peruvian immigrant parents settled in Chile and (2) to determine the possible influence of cultural sensitivity on the relationship between cultural diversity climate and parents’ school involvement. Results show that sociodemographic variables had limited effects on their school involvement, while cultural diversity climate, and cultural sensitivity had a greater influence. Implications for understanding immigrant parents’ relationships with schools and designing intervention programs are discussed.

## 1 Introduction

### 1.1 Parental school involvement

Parental involvement in children’s education has been extensively studied over the past decades, particularly regarding its impact on students’ positive development and academic success (Epstein, 2018). In fact, meta-analytic studies have indicated that parents’ participation in children’s school education predicts important outcomes such as school absenteeism and dropout (Gubbels et al., 2019), students’ social and emotional adjustment (Barger et al., 2019), as well as their academic achievement (Kim, 2022; Wilder, 2023). In this way, parental school involvement can have a key role in promoting their wellbeing and the development of positive life trajectories in the future.

There is a consensus on the importance of encouraging parents to actively support their children’s learning at home and participating in institutional and extracurricular school activities (Sandoval et al., 2017; Hoover-Dempsey et al., 2005). As such, parents’ engagement in children’s education has been understood as a type of social capital which allows them to benefit their children through their relationships with other parents and teachers (Murray et al., 2020; Turney and Kao, 2009). Involvement in education not only facilitates the transmission of the value of education but also provides parents with opportunities to access information and monitor their children’s school lives (Domina, 2005).

To better understand parental school involvement, Hoover-Dempsey and Sandler (1995, 1997) developed a model focusing on the psychological processes that determine it. This model stresses the relevance of parents’ motivational beliefs related to involvement, encompassing their role construction and self-efficacy in supporting their child’s academic success. A second key element is related to parents’ perceptions of involvement invitations from the school, which consist of specific requests to participate and the perception of a positive school climate. Besides, parents’ personal life contexts are additionally considered factors that shape their views on the feasible forms and timing of involvement, such as their skills, knowledge, available time, and energy for engagement.

In a more comprehensive manner, therefore, parental involvement could be understood as an emergent outcome of a complex system of relationships between multiple levels of the environment that influence each other. According to a socioecological perspective (Bronfenbrenner and Morris, 1998), school involvement occurs through the interaction between two systems: one at the microsystem, within the home, and the other at the mesosystem level, which is the school. The microsystem encompasses immediate relationships, daily activities, significant life events, and surrounding environments, which often serve as foundational reference points. As such, children undergo primary socialization at this level, with the microsystem playing a crucial role in shaping parenting practices and cultural transmission (Seginer, 2006).

On the other hand, the success (i.e., academic) resulting from home-school collaboration between parents and children has also been identified as an important factor for parents’ involvement development. Again, from a socioecological perspective, this second level (mesosystem) connects children to broader societal influences, such as parental involvement in school activities, which leads to parents becoming more engaged in their child’s education (Bronfenbrenner and Morris, 1998). Besides a third level, the exosystem, encompasses contexts that do not directly involve students but still impact their experiences in the immediate environment and include factors such as parents’ work conditions and health and welfare services, all supporting children’s development. Importantly, this perspective emphasizes a central aspect of the present study: the complex relation of factors that can affect parental involvement, which is most evident in immigration dynamics.

#### 1.1.1 Immigrant parents’ school involvement

Most of the available research on immigrant parents’ school involvement has been conducted in the United States, Europe, and Asia. In these contexts, it has been found low levels of participation in general, which, in turn, have been associated with parents’ low socioeconomic status, language barriers, and low academic expectations, as well as a presumed lack of interest in education (Calzada et al., 2015; Friedman et al., 2006; Li et al., 2020; González-Falcón et al., 2022). Moreover, it has been argued that lack of parental involvement could explain high rates of dropout and academic failure among immigrant students (González, 2022).

Likewise, the intersection of being an immigrant, low-income, and belonging to a cultural minority group altogether could lead to a more heightened mismatch of needs. This is because it increases the likelihood of schools successfully recognizing these parents’ academic knowledge and cultural backgrounds, hindering their participation (Antony-Newman, 2018; Ballenger, 2009; Calzada et al., 2015; Davis-Kean et al., 2021; Friedman et al., 2006; Ishimaru and Takahashi, 2017; Yu, 2020). Therefore, it may undermine parents’ perceived capabilities to contribute to their children’s academic progress by failing to recognize them as sufficiently qualified (Ballenger, 2009; Fang et al., 2017; Sheng, 2012). Moreover, it could negatively affect parents’ social identities and self-efficacy, hindering their willingness to actively participate in children’s schooling (Ballenger, 2009; Ishimaru and Takahashi, 2017; Yu, 2020). As a whole, this could affect parents’ construction of their role in their children’s education, which is developed through their relationship with the school and can be either facilitated or hindered depending on whether they are recognized as legitimate actors in transmitting knowledge (Ballenger, 2009; Rane and McBride, 2000).

Exacerbating this, cultural clashes between parents and school, as well as the perception of pressures to assimilate into the host culture, and repeated experiences of discrimination have been identified as important barriers to involvement for parents that belong to minority cultural groups (Onsès et al., 2023; Peček et al., 2008; Sime et al., 2017). In those cases, the educational centers could be perceived as a source of psychological and cultural threats, leading parents to limit their interactions with the school members in order to protect their cultural and personal identities (Antony-Newman, 2018; Marchand et al., 2019).

### 1.2 Explaining immigrant parents’ school involvement: cultural diversity climate and cultural sensitivity

#### 1.2.1 School cultural diversity climate

There have been identified several factors that parents consider crucial for their children’s school experiences. Among them are physical and psychological safety, the promotion of positive peer relationships, and support for the family (Ball et al., 2019; Baker et al., 2016; Hoover-Dempsey et al., 2005; Montecinos et al., 2010). The perception of a positive school climate, more specifically, appears particularly significant for parents from socially excluded groups (Baker et al., 2016; Reynolds et al., 2015), for whom school could represent a space of care and protection for their children (Davis-Kean et al., 2021). By establishing strong relationships and regular communication with educational institutions, parents can significantly influence their children’s academic outcomes and foster a more supportive and responsive school environment (Berkowitz et al., 2021; Koutsouveli and Geraki, 2022). This is because parental involvement is closely linked to the perceived positive recognition of their cultural identities within school communities among immigrant families (Hoover-Dempsey and Sandler, 1997; Hoover-Dempsey et al., 2005; Khalfaoui et al., 2020).

As one might expect, promoting a cultural diversity climate is a crucial aspect of the experiences of students, teachers, and families within multicultural schools (Bardach et al., 2024; Schachner et al., 2016; Schachner et al., 2019). Cultural diversity climate is defined as the strategies employed by schools to facilitate the coexistence of diverse cultural groups. These strategies are reflected in practices, norms, and behavioral guidelines that shape intergroup relationships among students from different ethnic backgrounds and between students and teachers (Baysu et al., 2024; Byrd, 2017; Schachner et al., 2019).

Approaches to cultural diversity in schools typically fall into two categories, which often underpin public policies in multicultural societies. The first approach focuses on preventing and reducing discrimination while promoting equity and inclusion for minority group members. The second approach emphasizes adherence to pluralism and the appreciation of cultural diversity as a resource for fostering harmonious coexistence and social cohesion (Chrobot-Mason and Aramovich, 2013; Fine-Davis and Faas, 2014; Schachner et al., 2016; Vandekerckhove and Aarssen, 2020). As such, cultural diversity reflects the extent to which schools implement these two approaches (Fine-Davis and Faas, 2014; Schachner et al., 2016; Schwarzenthal et al., 2020; Vandekerckhove and Aarssen, 2020). Numerous studies indicate that equitable treatment by teachers, along with opportunities for cooperation and shared goals among school community members, significantly contributes to reducing prejudice, discrimination, and intergroup conflict (Beelman and Heinemann, 2014; Cameron and Turner, 2017; Titzmann et al., 2015; Ülger et al., 2018).

On the other hand, promoting pluralism entails integrating respect and positive recognition of students’ cultural identities into teaching practices. In educational contexts, teachers tend to develop culturally sensitive pedagogies (Schachner et al., 2016) and create spaces for cultural exchange among families (Reynolds et al., 2015). The perception of equitable treatment, justice, and cultural recognition within the school environment has been linked to reduced discrimination, lower victimization rates, increased school participation, and enhanced wellbeing among minority group students (Özdemir and Stattin, 2013; Sirlopú and Renger, 2020). While scarce, available literature indicates that immigrant parents’ perceptions of how well their children’s and their own cultural identities are recognized and valued by schools is associated with parental involvement (Calzada et al., 2015; Baker et al., 2016; Khalfaoui et al., 2020; Reynolds et al., 2015; Sohn and Wang, 2006). Consequently, a supportive cultural diversity climate aligned with the type of cultural exchange that immigrant families seek could potentially enhance their engagement in school activities. In contrast, schools that predominantly adopt individualistic, assimilationist, or segregationist approaches may discourage immigrant parents from participating in their children’s education (Calzada et al., 2015; Baker et al., 2016; Khalfaoui et al., 2020; Reynolds et al., 2015; Sohn and Wang, 2006).

#### 1.2.2 Cultural sensitivity

Another important aspect to parental involvement is immigrant parents’ competencies to communicate in the school context. Usually studied under the name of cultural sensitivity, it is defined as the affective dimension of intercultural communication (Bennet, 1986; Chen and Starosta, 1998; Chen and Starosta, 2000). This competency involves the application of socio-cognitive skills to enhance awareness through intercultural interactions and the processing and comprehension of culturally relevant information. The development of cultural sensitivity extends beyond recognizing and understanding cultural differences and similarities; it also requires fostering positive attitudes toward individuals from diverse backgrounds, including accepting and respecting their cultural identities, thereby overcoming ethnocentric perspectives (Bhawuk et al., 2008).

These positive attitudes serve as motivators for engaging with outgroup members and enhance individuals’ willingness to adapt their behaviors in intercultural contexts (Chen and Starosta, 2000; Chao et al., 2017; Rania et al., 2012; Rodenborg and Boisen, 2013; Ting-Toomey, 2009). Furthermore, individuals who exhibit sensitivity in intercultural communication tend to engage more fully in such interactions and to experience enjoyment when meeting with members of other cultures (Chao et al., 2017; Chen and Starosta, 2000; Mera-Lemp et al., 2024; Zhang and Zhou, 2019). Additionally, it has been observed that these capabilities facilitate positive expectations about future interactions with members of other cultures, thereby increasing satisfaction with intergroup contact (Herrero-Hahn et al., 2019; Lee and Ma, 2019).

While cultural sensitivity can be an important resource for facilitating parental involvement in schools, it has received little attention in this context (Sohn and Wang, 2006). However, existing evidence indicates that, among immigrant students, these competencies are explained by the perception of positive contact norms and cooperation, which improves students’ intergroup attitudes, intercultural awareness, and the development of effective behavioral strategies in diversity scenarios (Schachner et al., 2015; Schwarzenthal et al., 2020). As these competencies increase, students’ satisfaction with their schooling and their integration within educational contexts improve (Mera-Lemp et al., 2020; Rania et al., 2012). In the same vein, research has reported that higher levels of intercultural sensitivity are associated with greater involvement of university students in both formal and informal academic activities within multicultural contexts (Tamam and Krauss, 2017). Several studies have also suggested that teachers’ cultural sensitivity promotes the development of pedagogical practices that include respect for and positive recognition of students’ cultural identities (Katıtaş et al., 2024; Mera-Lemp et al., 2024; Ulbricht et al., 2022).

### 1.3 The Chilean case

In Chile, the rapid increase in the immigrant population is leading to significant changes within the education system. As of 2022, foreigners constituted 8.3% of the total population, primarily originating from countries such as Venezuela (32.8%), Peru (15.4%), Colombia (11.7%), and Haiti (11.4%) (Instituto Nacional de Estadísticas, 2022). Consequently, immigrant students constitute 8% of overall national school enrollments, primarily concentrated in public schools (57.2%) and private subsidized institutions (38.1%) (Servicio Jesuita a Migrantes, 2024).

Official data indicate that the living conditions of immigrant families are often precarious. Despite their increased presence in the workforce, immigrants typically earn lower incomes, have lower rates of community participation, and have limited access to support networks compared to native citizens (Ministerio de Desarrollo Social y Familia, 2024). Immigrant families are highly exposed to discrimination based on their cultural origins and encounter more significant barriers to accessing social services and healthcare than Chileans (Aninat and Vergara, 2020; Cabieses and Oyarte, 2020; Grau et al., 2021). In the same vein, more than 26% of immigrant children and adolescents aged 1–18 live in conditions of multidimensional poverty, a rate that double that of the Chilean population (Servicio Jesuita a Migrantes, 2021).

Immigrant students are also concentrated in schools which are often characterized by high levels of vulnerability, low educational quality, and school climate problems (Eyzaguirre et al., 2019). Additionally, results from PISA test have shown that these students tend to present lower levels of academic achievement than Chileans (Agencia de Calidad en Educación, 2022), which coincides with the international tendency (Karakus et al., 2023). Besides, they exhibit lower attendance rates compared to their native counterparts across all levels of education (García and Friz, 2019).

Although the Chilean education system is implementing changes to address these sociocultural transformations by promoting inclusion and intercultural teaching, research has revealed difficulties in managing social and cultural diversity within schools (González et al., 2023; Ortega et al., 2020). For instance, different studies have shown the existence of intergroup conflicts, stressing the negative effects of discrimination and prejudice on their school experiences (Guthrie et al., 2019; Mera-Lemp and Martínez-Zelaya, 2021; Pavez-Soto et al., 2019; Segovia-Lagos et al., 2023).

Taken together, these antecedents indicate that immigrant families settled in Chile are facing several difficulties in their relationships with schools, which seem to be leading children to an important academic disadvantage. Given the impact of experiences at school and academic achievement on children’s positive development as well as in their social and cultural integration in the future, it is important to consider the role of their parents as key actors during their schooling processes (Boonk et al., 2018).

#### 1.3.1 Immigrant parents in the Chilean educational system

While the overall research on immigrant parents’ experiences in Chilean schools is limited, past research examining the perspectives of Chilean teachers highlights the negative implications of insufficient training in culturally responsive teaching practices, as well as the perception of cultural threat and biases toward immigrant students and their families (Flanagan-Bórquez et al., 2021; Riedemann et al., 2020). Additionally, some studies have indicated that native teachers often exhibit ambivalence when assessing immigrant parents’ abilities to take an active role in their children’s education, suggesting that these parents do not sufficiently engage in school activities (Segovia and Rendón, 2020).

On the other hand, research on acculturation preferences indicates that Latino immigrant adults and adolescents strongly desire to maintain their original cultural identities while incorporating elements of Chilean culture. This dual commitment is linked to higher levels of wellbeing and social connectedness with host society members (Mera-Lemp et al., 2021; Mera-Lemp and Martínez-Zelaya, 2021). Nonetheless, despite these parents wanting their children to maintain their cultural identities and learn about Chilean culture, they often promote cultural assimilation at school to avoid conflict (Oyarzún et al., 2022). Additionally, immigrant parents prefer enrolling their children in schools with a significant immigrant population to minimize their exposure to discrimination from native students (Eberhard and Lauer, 2019). This suggests that parents’ participation could be hindered by difficulties in their interactions with teachers and Chilean parents (Joiko and Vásquez, 2016).

### 1.4 Objectives and hypotheses

On summary, prior research suggests that the intercultural sensitivity of immigrant parents may be explained by the extent to which they perceive a climate of cultural diversity that facilitates positive intergroup relations at school settings (Sohn and Wang, 2006). Furthermore, feeling capable of communicating and sharing culturally based knowledge within the school environment can enhance their engagement in school activities, thereby supporting the positive development of their children (Swap, 1993). Based on these antecedents, this study aims (1) to study the relationships between cultural diversity climate, cultural sensitivity and school involvement perceived by Latin-American immigrant parents settled in Chile, and (2) to the determinate the possible influence of cultural sensitivity on the relationship between cultural diversity climate and parents’ school involvement. As a hypothesis, we expect that: 1. Parental school involvement will be explained by cultural diversity climate; 1.1. Parental school involvement will be explained by cultural sensitivity; 2. Cultural sensitivity will mediate the effects of cultural diversity climate on parental school involvement.

## 2 Materials and methods

### 2.1 Participants and procedure

The present study uses a correlational design and a non-probabilistic convenience sample of immigrant parents and tutors of school children The sample was collected by professional interviewers of immigrant origin who went to the areas surrounding the educational establishments (*k* = 171 schools) in 31 districts of the city of Santiago to invite immigrant school guardians. In turn, study participants could in addition recommend people they knew to participate, and the main inclusion criteria were (a) being an immigrant of Peruvian or Venezuelan origin and (b) being a parent of a student in grades 1–4 in an educational establishment in Santiago.

**Figure 1:**
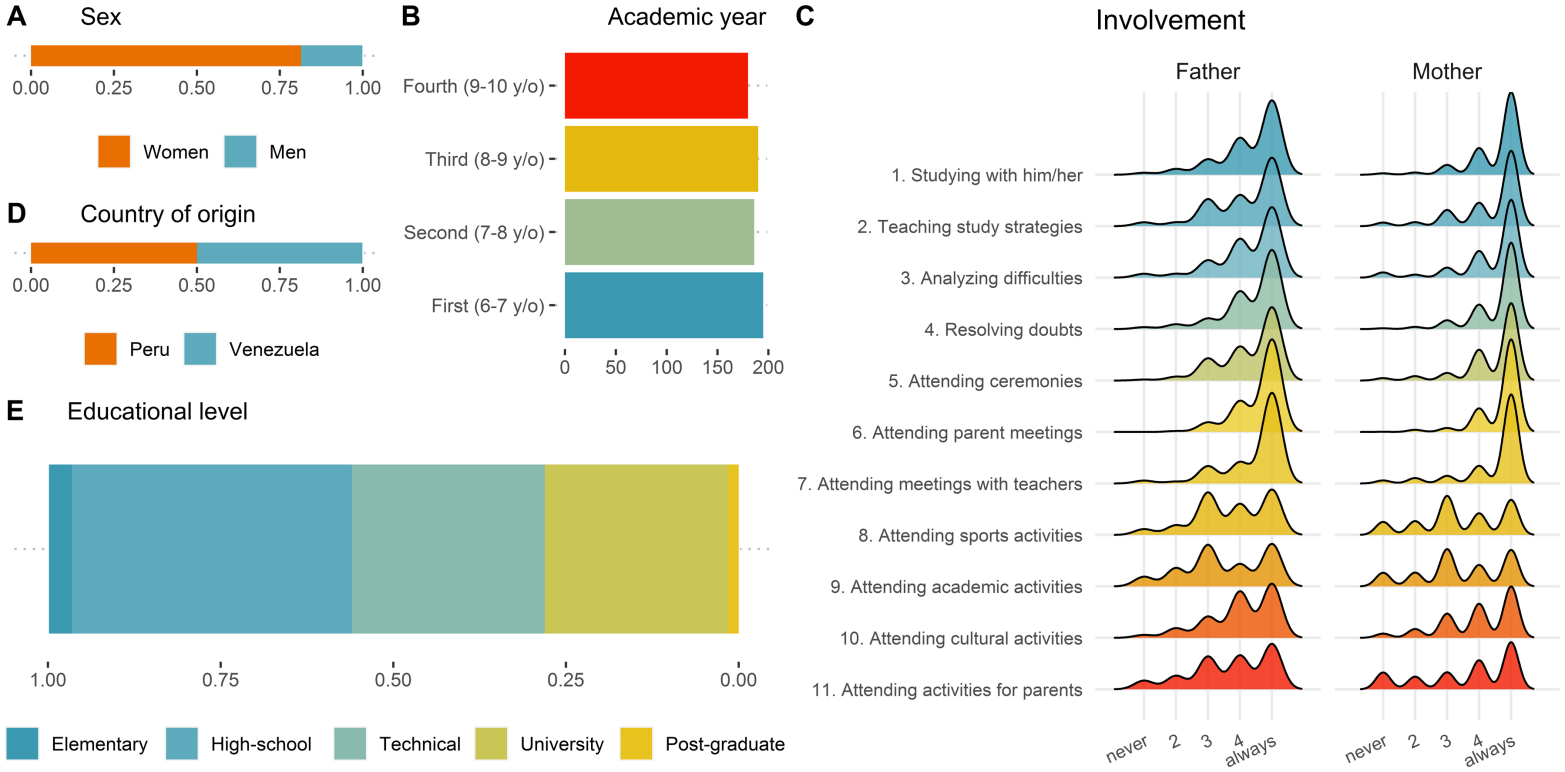
Descriptive information of the sample.

The final sample consisted in 751 participants (visual descriptions in Figure 1), 18.5% were males and the other 81.4% females, with no differences in the proportion their proportion by country of origin (*χ*^2^(1) = 0.456; *p* = 0.500). They were equally from Peru and Venezuela and their pupils were in first (6–7 years) to fourth grade (9–10 years) of the Chilean educational system. Participants’ consent was requested, aiming to safeguard voluntarily participation. The procedures followed in the study were certificated by the Research Ethics Committee of Universidad Alberto Hurtado, considering all the standards of the Helsinki Declaration.

### 2.2 Variables and instruments

Each participant answered demographic information regarding their gender, age, nationality, educational level, length of stay in Chile, type of school attended by their student and length of time as a parent or guardian. Besides, they were asked to inform their child’s gender and academic year. In addition, they answered the following measures:

#### 2.2.1 Cultural diversity climate

We adapted 18 items from the Affirming Climate for Workplace Diversity (Chrobot-Mason and Aramovich, 2013). This scale was originally created to assess immigrants’ perceptions of the cultural diversity climate within organizations, and its validation (i.e., construct and criterion) was carried out with a large sample of public servants (Chrobot-Mason and Aramovich, 2013). In this study, it was used to measure parents’ perceptions of equitable treatment, equal access to opportunities and identification with the institution, and identity recognition (e.g., *“In the school where I am a guardian, immigrants receive fewer opportunities than Chileans (scholarships, social assistance, support for guardians and students, etc.,” “In the school where I am a guardian, both Chileans and immigrants are treated fairly”*) on a Likert scale from 1 (Very disagree) to 4 (Very agree). In the present study, total reliability was of Alpha = 0.93.

#### 2.2.2 Cultural sensitivity

The cultural sensitivity scale (Chen and Starosta, 2000), validated in Chile by Martínez-Zelaya et al. (2020), was applied. It consists of 24 Likert-type items with four answer options (1 = Very disagree, 4 = Very agree) (e.g., “*When I speak with people from different cultures, I try to gather as much information as possible*,” “*I enjoy being with people from different cultures*”). Previous studies have successfully analyzed its construct and criterion validity (Martínez-Zelaya et al., 2020; Lahoz i Ubach and Cordeu Cuccia, 2021). In this study, the scale’s reliability was alpha = 0.91.

#### 2.2.3 Parental school involvement

Eleven items from the School Involvement Questionnaire of the Metropolitan Survey on Family and Education, Centre for Family Studies and Research (CEIF) of Universidad Finis Terrae, were applied. These items measured the frequency in which parents participate at school, including both academic (e.g., “*Meetings with teachers*”) and extracurricular activities (e.g., “*Sports activities organized by the school*”), as well as the extent in which they support their children at home (e.g., “*To study with this child for tests*”). This scale has been used previously in Chilean studies, which have effectively examined its construct and criterion validity (i.e., Gubbins and Otero, 2018; Gubbins and Otero, 2020). In this research, it presented Alpha: Home = 0.90; School = 0.87; Total = 0.88.

Finally, the official number of immigrants in the commune obtained through the National Institute of Statistics (Instituto Nacional de Estadísticas, 2022) was included as collective level data in relation to the communes where participants were surveyed.

### 2.3 Analyses

First, we performed a series of confirmatory factor analyses to evaluate the psychometric properties of the scales, with their respective reliability tests (i.e., Cronbach’s Alpha). For testing the main hypotheses, we conducted multi-level modeling and plotted the main results and finally, we evaluated a mediational model to test our hypotheses. All analyses were conducted in R (R Core Team, 2014) with RStudio (RStudioTeam, 2015). For reliability analysis and the mediational model, we used the packages lavaan (Rosseel, 2012) and semTools (Jorgensen et al., 2022). For correlations, apaTables (Stanley and Spence, 2018) was used. For multilevel models, lme4 (Bates et al., 2015), and sjPlot (Lüdecke, 2020) for tables and multilevel moderation effects. Finally, we used the package wesanderson for colors (Ram and Wickham, 2023). All the data and the syntax with all the analyses supporting our main analyses are made available in our Supplementary material section.

## 3 Results

The correlation matrix (Table 1)^1^ revealed a negative association between sex and educational level, suggesting that men have marginally lower education levels than women and school involvement at home, meaning that females tend to be slightly more involved in school-related tasks at home than males. Further significant associations were found between Nationality and Stay in the Country, indicating that Peruvians with pupils at school (compared to Venezuelans) tended to stay more in Chile. Finally, educational level had a strong negative relationship with Nationality, indicating that, for this particular sample, Peruvians had levels of education compared to Venezuelans, which was further corroborated with a Chi-square test (*χ*^2^(4) = 134.86; *p* < 0.001).

**Table 1:** Means, standard deviations and correlations among variables.

Variable	*M*	*SD*	1	2	3	4	5	6	7	8	9	10	11	12
1. Sex^1^	–	–												
2. Age	35.82	7.27	−0.10**											
			[−0.17, −0.02]											
3. Nationality^2^	–	–	0.02	0.02										
			[−0.05, 0.10]	[−0.05, 0.09]										
4. Ed. Level	2.82	0.91	−0.10**	−0.01	−0.36**									
			[−0.17, −0.03]	[−0.08, 0.06]	[−0.42, −0.30]									
5. Stay	7.45	5.61	−0.06	0.21**	0.64**	−0.25**								
			[−0.13, 0.01]	[0.14, 0.28]	[0.59, 0.68]	[−0.32, −0.18]								
6. Dependency^3^	–	–	−0.05	0.09*	0.00	0.11**	0.05							
			[−0.12, 0.02]	[0.02, 0.16]	[−0.07, 0.07]	[0.04, 0.18]	[−0.02, 0.12]							
7. T. Guardian	2.89	1.97	−0.03	0.24**	0.31**	−0.11**	0.48**	0.06						
			[−0.10, 0.04]	[0.18, 0.31]	[0.24, 0.37]	[−0.18, −0.03]	[0.42, 0.53]	[−0.02, 0.13]						
8. Aca. Year	2.47	1.12	−0.03	0.18**	0.07*	0.02	0.11**	−0.01	0.25**					
			[−0.10, 0.04]	[0.11, 0.25]	[0.00, 0.14]	[−0.05, 0.09]	[0.04, 0.18]	[−0.08, 0.06]	[0.18, 0.31]					
9. CSC	3.20	0.69	−0.08*	−0.03	−0.01	−0.02	0.03	0.01	0.13**	0.02				
			[−0.15, −0.01]	[−0.10, 0.05]	[−0.08, 0.06]	[−0.09, 0.06]	[−0.04, 0.10]	[−0.06, 0.08]	[0.06, 0.20]	[−0.06, 0.09]				
10. CS	3.87	0.49	−0.05	0.04	−0.22**	0.10**	−0.11**	0.04	0.02	0.02	0.38**			
			[−0.13, 0.02]	[−0.04, 0.11]	[−0.29, −0.15]	[0.02, 0.17]	[−0.18, −0.04]	[−0.03, 0.11]	[−0.05, 0.09]	[−0.05, 0.09]	[0.32, 0.44]			
11. Involv. H.	4.42	0.81	0.08*	−0.01	−0.07*	0.08*	−0.04	−0.02	0.03	−0.13**	0.18**	0.19**		
			[0.01, 0.15]	[−0.08, 0.06]	[−0.15, −0.00]	[0.00, 0.15]	[−0.11, 0.03]	[−0.09, 0.05]	[−0.04, 0.10]	[−0.20, −0.06]	[0.11, 0.25]	[0.12, 0.26]		
12. Involv. S.	4.02	0.83	−0.02	0.08*	0.04	0.11**	0.08*	0.17**	0.18**	0.00	0.23**	0.19**	0.42**	
			[−0.09, 0.05]	[0.01, 0.15]	[−0.03, 0.11]	[0.04, 0.18]	[0.00, 0.15]	[0.10, 0.24]	[0.11, 0.25]	[−0.07, 0.07]	[0.16, 0.29]	[0.12, 0.25]	[0.36, 0.48]	
13. Involv. T.	4.16	0.70	0.02	0.06	−0.00	0.11**	0.04	0.12**	0.15**	−0.05	0.24**	0.22**	0.73**	0.93**
			[−0.05, 0.09]	[−0.02, 0.13]	[−0.07, 0.07]	[0.04, 0.18]	[−0.03, 0.11]	[0.05, 0.19]	[0.07, 0.21]	[−0.13, 0.02]	[0.18, 0.31]	[0.15, 0.29]	[0.70, 0.77]	[0.91, 0.93]

*M* and SD are used to represent mean and standard deviation, respectively. Values in square brackets indicate the 95% confidence interval for each correlation. ^1^1 = Men, 2 = Women. ^2^Nationality is the comparison between immigrants from Venezuela (1) and Peru (2). ^3^Dependency indicates whether each school was public (i.e., fully maintained by the state; 1) or subsidized (i.e., financed by the state but administrated by private organizations; 2). CDC = Cultural Diversity Climate; CS = Cultural Sensitivity. Involv. H., Involv. S., and Involv. T., indicate Involvement in School with tasks at Home, School and the Total score, respectively. *p < 0.05. **p < 0.01.

**Figure 2:**
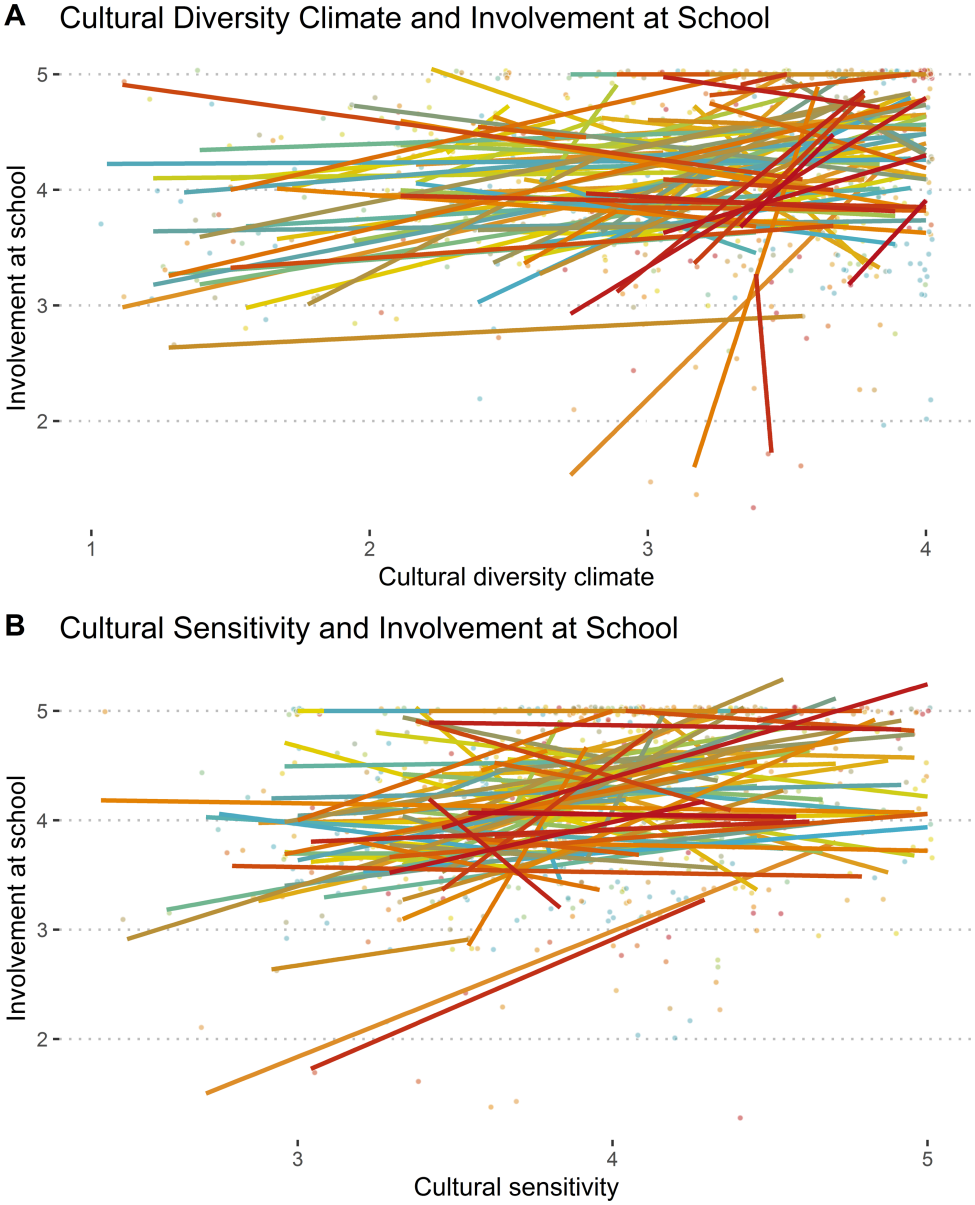
Linear relations between the variables. Each line represents a linear regression predicting involvement at school by either cultural diversity climate **(A)** or cultural sensitivity **(B)**, across 171 schools of Santiago, Chile.

Before conducting the main analyses, we evaluated the true nature of the measured constructs (i.e., Culture Diversity Climate and Involvement at School, and the former with Cultural Sensitivity, as seen in Figures 2A,B, respectively). As Figure 2A shows, some lines suggest a positive relationship, while others remain flat or neutral, or even negative. Similarly, in plot B the pattern of lines also shows a diverse set of relationships, with many lines showing minimal slope, although some suggest a slight positive trend between the constructs. As a whole, these visual analyses suggest that the true nature of the variables is nested in schools and therefore, we will test the main hypotheses through multilevel regressions.

As can be seen in the analyses (Table 2), the models explained a substantial percentage of the variance in school involvement, the largest being related to school activities. In detail, among the variables that explained total school involvement is the fact of being female (vs. male), having a higher educational level, having more years as parents and that the pupil is from a younger grade. Finally, the variables that explain the most variance are higher levels of cultural diversity climate, on the one hand, and cultural sensitivity, on the other.

**Table 2:** Multilevel models predicting involvement at school across 171 schools in Santiago.

Predictors	Total score			Home			School		
	*β*	95% *CI*	*p*	*β*	95% *CI*	*p*	*β*	95% *CI*	*p*
(Intercept)	0.03	−0.07, 0.13	<0.001	−0.02	−0.11, 0.08	<0.001	0.06	−0.05, 0.16	<0.001
Sex^1^	0.09	0.02, 0.15	0.008	0.11	0.05, 0.18	0.001	0.05	−0.01, 0.12	0.094
Age	0.02	−0.04, 0.09	0.483	0.00	−0.07, 0.07	0.995	0.03	−0.03, 0.10	0.331
Nationality^2^	0.01	−0.10, 0.12	0.849	−0.05	−0.17, 0.07	0.440	0.04	−0.06, 0.14	0.442
Edu. Level	0.10	0.02, 0.17	0.009	0.08	0.00, 0.15	0.047	0.09	0.02, 0.16	0.014
Stay	0.00	−0.09, 0.10	0.987	−0.01	−0.11, 0.09	0.876	0.00	−0.09, 0.09	0.946
Dependency	0.10	−0.00, 0.20	0.053	−0.04	−0.13, 0.05	0.385	0.13	0.03, 0.23	0.010
Time guardian	0.10	0.02, 0.18	0.011	0.03	−0.06, 0.11	0.539	0.13	0.05, 0.20	0.001
Academic year	−0.07	−0.14, −0.01	0.035	−0.11	−0.18, −0.03	0.004	−0.05	−0.11, 0.02	0.182
CDC	0.18	0.10, 0.25	<0.001	0.13	0.05, 0.21	0.001	0.17	0.09, 0.24	<0.001
CS	0.14	0.07, 0.22	<0.001	0.12	0.04, 0.20	0.003	0.13	0.05, 0.20	0.001
Immigrants^3^	−0.03	−0.12, 0.05	0.450	−0.05	−0.15, 0.04	0.262	−0.01	−0.09, 0.08	0.904
Random effects									
σ2	0.36			0.52			0.47		
τ00 School	0.29			0.33			0.34		
τ11 School Nationality	0.05			0.16			0.02		
ρ01 Centro	−0.89			−0.93			−1.00		
*ICC*	0.21			0.15			0.22		
*N* Centro	171			171			171		
Observations	751			751			751		
Marginal *R*^2^/Conditional *R*^2^	0.112/0.296			0.074/0.210			0.114/0.313		

^1^1 = Men, 2 = Women. ^2^Nationality is the comparison between immigrants from Venezuela (1) and Peru (2). ^3^The variable represents the number of immigrants in the commune within Santiago. CDC = Cultural Diversity Climate; CS = Cultural Sensitivity. Involv. H., Involv. S., and Involv. T., indicate Involvement in School with tasks at Home.

**Figure 3:**
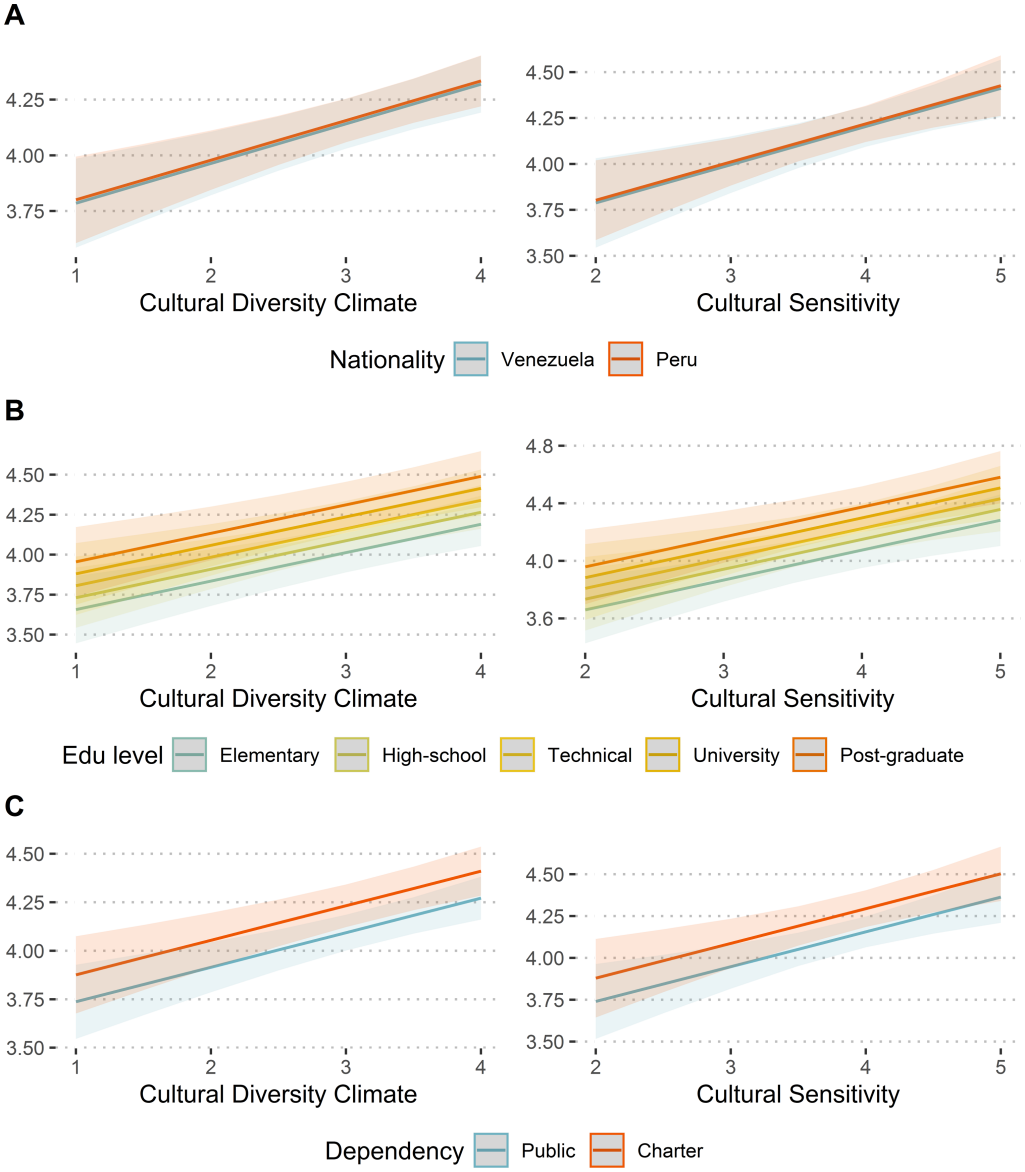
Effects of cultural diversity climate and cultural sensitivity on involvement across levels of demographic variables. Standardized multilevel coefficients from the models predicting involvement (total score). The figure separates the effects of the main predictors on the dependent variable across levels of nationality **(A)**, educational level **(B)**, and the type of school **(C)**. Shaded areas represent the 95% confident intervals.

In a detailed analysis, examining activities at home and activities at school, it is observed that both the climate of cultural diversity and cultural sensitivity are the variables that most explain the dependent variables. In addition, it is seen that gender (i.e., being female vs. being male) explains only home activities, and that time as a proxy explains only school activities. On the other hand, educational level significantly explains both variables, while dependence (i.e., public or private and subsidized school) only explains greater involvement in school activities. In none of the cases is there any effect of nationality, age or length of stay in the country. Additionally, we evaluated whether the effects of the cultural diversity climate and cultural sensitivity were affected by their interaction with participants’ nationality, educational level, or the type of school (Figure 3). As can be seen, in all cases, there were positive effects of both cultural diversity climate and cultural sensitivity regardless of the levels of these variables.

Finally, we tested a simple mediation model to explain the scores of the two facets of Involvement in School as it is seen in Figure 3. The overall fit of the path analysis revealed an excellent adjustment (*CFI* = 1.000, *TLI* = 1.000, *RMSEA* = 0.000, 95% CI [0.00, 0.00]) and revealed that there are indirect effects of Cultural Diversity Climate on the two facets of Involvement. In both cases, higher scores in Cultural Diversity Climate, through greater Cultural Sensitivity, explained greater Involvement in home activities (*β* = 0.06; SE = 0.02, 95% CI [0.03, 0.10]) and in school activities (*β* = 0.04; SE = 0.02, 95% CI [0.02, 0.09]).

## 4 Discussion

**Figure 4:**
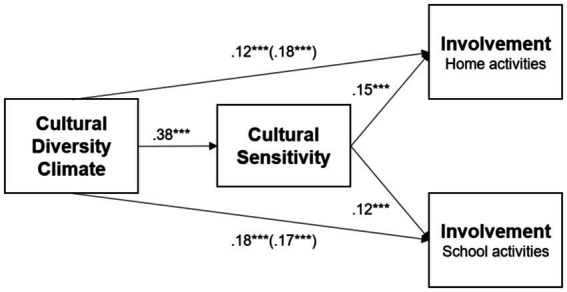
Mediation effects of cultural diversity climate on parental involvement. Values represent standardized regression coefficients among the variables; values between brackets indicate the total model effects model for each criterion variable. ***p < 0.001.

This study shows that immigrant parents’ school involvement is shaped by the interaction of different levels of their experiences, such as home, school, and sociocultural factors, as has been stated by several authors (i.e., Antony-Newman, 2018; Bronfenbrenner and Morris, 1998; Hoover-Dempsey and Sandler, 1997). Particularly, our results support the idea that cultural diversity climate and cultural sensitivity are fundamental aspects of immigrant parents’ involvement at Chilean schools. It also shows that institutions that create favorable conditions for intergroup contact through practices of positive recognition of cultural identities, equitable treatment and equal access to opportunities (for both Chilean and immigrant parents), foster immigrant guardians’ commitment. Importantly, this includes not only parental participation in school activities but also encourages their engagement in helping children with their learning processes at home (Figure 4).

The results presented here indicate that, when schools positively manage cultural diversity, parents’ socio-cognitive skills and attitudes to communicating in intercultural settings are enhanced. In turn, cultural sensitivity promotes greater involvement in their children’s education. Higher skills for interacting with native teachers and parents may facilitate intergroup contact and social performance in the case of our participants. Interestingly, our findings show that they also influence the extent to which these parents are involved in their children’s school education at home, suggesting that cultural sensitivity could lead to a better attitude toward the culturally based school content that children must learn and study at home. Remarkably, the effects of sociodemographic variables (i.e., gender, age, educational level, length of stay in the country) only had a minor influence on parents’ involvement despite being focused on in past research. Together with the fact that the present study’s sample was composed of participants of the nationalities representing the two main immigrant communities in Chile, these findings highlight the relevance of cultural diversity climate and cultural sensitivity. Indeed, these variables can be considered assets that could improve immigrant parents’ involvement with diverse backgrounds and at different contexts such as public or subsidized schools with varying levels of immigrant population sizes.

These outcomes are especially relevant because immigrant parents find themselves in a situation of significant disadvantage compared to the majority group. On the one hand, they must overcome several barriers to access basic social welfare conditions. Furthermore, they belong to social categories with low recognition, status and power, exposing them to different forms of discrimination. The fact that cultural diversity climate, as well as cultural sensitivity, tend to have a greater influence on parental involvement than sociodemographic variables, such as educational level, suggests that schools have the potential to become spaces that can legitimize immigrant parents by ensuring equality and justice in their relationships. Certainly, educational institutions cannot modify the structural conditions that constrain these parents’ participation in children’s schooling. However, by improving parental involvement, schools can promote students’ social inclusion by fostering their academic achievement and positive development. When educational centers do not provide the necessary conditions for students to expand their personal, social, and cultural resources, they reproduce social inequalities, particularly affecting those from minority groups (Ali, 2008; Mzidabi et al., 2024).

In fact, several authors (Bankston, 2004; Martínez-Taboada et al., 2017; Ogbu and Simons, 1998; Olmedo, 2003) have indicated that lack of recognition, pressure to assimilate, and unfair treatment can lead to cultural resistance strategies, resulting in absenteeism, academic failure, and school dropout. These problems are often experienced by immigrant children, who usually are at high risk of poor academic results and frequently live in vulnerable conditions (Alieva et al., 2024; Özdemir and Bayram Özdemir, 2020) and might keep families from minority groups in precarious situations, perpetuating dynamics of exclusion and social marginalization (García, 2011). Strong collaboration and egalitarian relationships between parents and school staff could help to reverse these cycles of inequality (Khalfaoui et al., 2020; Serna et al., 2008).

Given the substantial challenges that educational systems encounter because of the multiple needs of their students and families, it seems that improving the cultural diversity climate and enhancing parents’ cultural sensitivity could have high cost–benefit potential. According to our results, future interventions could focus on establishing school policies that guarantee equal treatment and opportunities, and respect for cultural identities. This could also help teachers and administrators improve their attitudes toward multiculturality and intergroup relationships between students. Besides, intercultural communication competencies training programs for parents at schools could strengthen their participation in children’s education and reinforce their commitment to the institutions. Also, it could facilitate their integration into the host society in different dimensions of their lives.

### 4.1 Parents’ school involvement, cultural diversity climate and cultural sensitivity perceptions

We have found high levels of cultural diversity climate, intercultural sensitivity, and additionally, involvement in school in the present sample, and several reasons could explain this. First, heightened levels of intercultural sensitivity align with reports from immigrant secondary education students in Chile (Lahoz i Ubach and Cordeu Cuccia, 2021). Even though they have operationalized cultural competence in a different manner, other local studies with immigrant students (Mera-Lemp et al., 2020), have shown notable capabilities in both understanding information from the local culture and interacting with native peers in school. This has been attributed to the fact that immigrants must constantly confront the task of navigating information from a new culture, as well as communicating with members of the host society, which may facilitate the development of these competencies (Mera-Lemp et al., 2020; Rania et al., 2012).

Second, high levels of school involvement, both in the home context and through direct participation in school activities, may be linked to higher levels of collectivistic cultures; specifically, due to the relevance of societal norms and conformity (see Henrich, 2020). In fact, families from more collectivistic cultures often adopt parenting styles that emphasize the importance of duty fulfillment and obedience, thereby promoting behavioral control (Bornstein, 2017; Bornstein and Cote, 2006; Gallardo, 2019). Furthermore, it has been suggested that immigrant parents from low-income countries are particularly concerned with transmitting to children the values of hard work and discipline and recognizing the role of education in facilitating social mobility (Nesteruk and Marks, 2011).

It is important to note that these results differ from previous studies, which suggested that immigrant parents in Chile perceived tensions in their relationships with the schools (Eberhard and Lauer, 2019; Oyarzún et al., 2022). This could be attributed to the fact that this study focused on parents’ perceptions about their own relationships with school agents rather than the practices implemented within the classroom context. Furthermore, methodological differences could explain these discrepancies, such as using quantitative methodology and including a wide diversity of schools. Besides, research on acculturation attitudes within the Chilean population has described a strong inclination toward individualism. This suggests that Chileans often interact with immigrants as individuals with distinct characteristics and personal goals rather than emphasizing group affiliations (Martínez-Zelaya et al., 2020; Mera-Lemp et al., 2021). Such an approach may contribute to the perception of equitable and fair treatment by teachers and administrative staff.

Finally, recent studies have also reported that even though Chilean teachers have had to face the challenges of teaching immigrant students with little knowledge about intercultural education and scarce resources, it seems that their attitudes toward multiculturality at school tend to be positive (Mera-Lemp et al., 2024). It is possible that the experience of continuously teaching immigrant students is leading them to new insights, which could boost the development of practices such as the incorporation of content based on the students’ cultures of origin and creating strategies to establish trust-based relationships with immigrant families (Mendoza Mardones, 2024).

### 4.2 The role of sociodemographic variables

Analyzing the relevance of sociodemographic variables, we found that mothers are more prone (compared to fathers) to involve themselves in supporting children in homework and studying at home. In addition, in the present sample, fathers indicated lower levels of cultural diversity at schools than mothers. As is widely recognized, parental roles in children’s education are often shaped by parents’ expectations and their cultural contexts (Hoover-Dempsey and Sandler, 1997), and these expectations are inherently gendered. Thus, mothers tend to deal with stronger role expectations than fathers regarding active daily involvement, which could lead to a great engagement in the home context (Kim and Hill, 2015; McBride et al., 2009). In the same vein, and while there is a lack of evidence regarding the role of gender in the perception of the school diversity climate, some studies have suggested that women tend to perceive discriminatory attitudes to a lesser extent than men due to cognitive biases that allow them to pay less attention to, or even deny, unfair treatment. This could explain why fathers perceived, to a lesser degree, that the school provides equal treatment and equal access to opportunities compared to women (Kim and Noh, 2014).

Nonetheless, this hypothesis (i.e., differential perception of discriminatory attitudes) is not the only possible explanation since there are no differences between genders in parental involvement at school, and the differences between fathers/mothers in school involvement at home are small in magnitude (see also Figure 1C). While the literature has focused on school involvement among women, the absence of differences by gender in the present study could be seen as a positive outcome, as there is evidence highlighting the importance of fathers’ participation for the educational trajectories and wellbeing of their children (Lazović et al., 2022; Rollè et al., 2019). On the other hand, there were no differences between Venezuelan and Peruvian parents regarding their perceptions of cultural diversity climate (see Figure 3A). Nonetheless, Venezuelan participants seem to present higher levels of cultural sensitivity and a stronger involvement at home. This could be because Venezuelan participants presented higher levels of education, and yet, parents’ nationality was not related to any of the other variables.

While parents’ educational level was not related to the perception of cultural diversity climate, it was positively associated with cultural sensitivity; at least in intensity (see Figure 3B). Some studies (Sari and Yalçinkaya, 2023) have suggested that academic education contributes to a more positive attitude toward members of other cultures, as well as to an increased ability to communicate in multicultural environments. This also supports the idea that parents with higher cultural capital would be more likely to actively participate in children’s education, possibly because their own academic knowledge is aligned with what is promoted by the school (Antony-Newman, 2018; Bourdieu and Passeron, 1990; Epstein, 2018; Hornby and Lafaele, 2011). This underlines the importance of promoting the involvement of those parents for whom a low educational level intersects with being immigrants, placing them in a disadvantaged position (Fang et al., 2017; Sheng, 2012).

Parents’ length of stay in Chile was not related to cultural diversity climate perception, probably because the quality of the relationship with the school plays a greater role than the extent of the experience at the receiving country, as has been previously observed in samples of immigrant students (Mera-Lemp et al., 2020). Strikingly, however, it was found that the longer its’ duration, the lower their cultural sensitivity. Despite this could be considered counterintuitive, acculturation processes could indeed affect this competence. For example, it is possible that, as immigrants understand and learn the host society’s culture, socio-cognitive efforts during intercultural communication decrease. On the contrary, the development of stronger attachments to their own cultural background could decrease their sensitivity (Martínez-Zelaya et al., 2020). Sustained emotional negative experiences through intergroup contact can also diminish immigrants’ intercultural competencies (Mera-Lemp et al., 2020).

Conversely, time in the country had a positive relationship with involvement at school activities, suggesting that greater familiarity with the local culture facilitates their participation in school. Furthermore, the longer parents are connected to the school, the greater their perception of the cultural diversity climate, as well as their involvement at home and in the school. Understanding institutions, along with the establishment of relationships with their members over time, would facilitate the perception of belonging, fairness, and recognition of one’s own cultural identity at school. It is also possible that school agents tend to establish interpersonal relationships with immigrant parents over time, placing less emphasis on belonging to different intergroup categories. The academic year in which the children were enrolled was only associated with parental home involvement, indicating that the younger the children are, the greater the parental participation in their education at home is (Gubbins and Otero, 2018).

In all, sociodemographic variables cannot fully explain the levels of parental involvement in this large and heterogeneous sample; rather, as we expected, cultural diversity climate and cultural sensitivity are those that more effectively explain it. Additionally, their effects are kept invariant across levels of some of these sociodemographic variables. These results reinforce the relevance of generating positive conditions for intergroup contact at school and the important role of parents’ intercultural competencies.

### 4.3 Limitations, strengths and future research lines

This study has limitations that should be considered. First, using an intentional sample in the framework of a cross-sectional design does not allow us to appreciate the development of the relationship between the studied variables. It could be interesting to study cultural sensitivity’s trajectories explaining parental school involvement across time. Additionally, a potential future direction would involve testing this through an experimental design within the context of a training program focused on competencies to assess how it may (or may not) explain parental school involvement.

Second, although our participants come from countries with significant representation in Chile, it would be important to examine whether the variables of this study can also explain parents’ involvement in a more heterogeneous sample of immigrant groups. This would allow for testing, among other factors, cultural explanations (e.g., levels of collectivism or relative differences in individualism) within these samples. Third, this research only considers parents’ reports without including teachers’ and students’ points of view. Even though there are scarce antecedents of parental school involvement in Chile, our results seem to be consistent with recent studies that have shown that teachers’ attitudes toward multiculturality at school tend to be positive (Mendoza Mardones, 2024; Mera-Lemp et al., 2024), which could be facilitating parents’ experiences at school. Also, some studies conducted with immigrant students in the local context have found high levels of school satisfaction (Mera-Lemp et al., 2020) and positive school climate perceptions (Lahoz i Ubach and Cordeu Cuccia, 2021). However, future studies with immigrant parents should consider the importance of including pupils and school staff to carry out data triangulation.

Notwithstanding, this work contributes to comprehending immigrant parents’ school involvement in the context of South–south migration, which has been very scarcely studied. Besides, and despite its relevance, parents’ perception of the cultural diversity climate at school and its effects have received little attention, so this study represents a contribution to developing this research line. Finally, it is important to remark that our results suggest intervention perspectives with significant cost–benefit potential for schools. Considering the great impact that the increase of migratory flows is having on the educational systems, this could be an interesting alternative to explore.

## 5 Conclusion

This study shows two psychological variables can influence the school involvement of immigrant parents in Chile: the school’s cultural diversity climate and cultural sensitivity. In those institutions where equal treatment, recognition of different cultural identities, and social inclusion are fostered, environments are created that encourage immigrant parents to become involved not only in school activities at school but also in learning at home. Although involvement also depends–to a lesser extent– on sociodemographic variables (e.g., gender, educational level or academic year), these results highlight the importance of an inclusive school climate that supports intergroup relations and parental participation. Considering the benefits of parental involvement in school on academic success or social–emotional wellbeing, educational public policies could prioritize interventions that improve the climate of cultural diversity and promote intercultural competencies in both parents and educational staff.

## Data Availability

The original contributions presented in the study are included in the article/Supplementary material, further inquiries can be directed to the corresponding author.

## Footnotes

^1^We consider it important to mention that, due to the nature of the data in this research, the correlation table should be taken as a guideline and not as a reflection of what actually occurs between the variables.

## References

[ref9001] Agencia de Calidad en Educación. (2022). Informe Nacional PISA 2022. Available online at: https://hdl.handle.net/20.500.12365/20287pdf (Accessed October, 21, 2024).

[ref1] Ali M. A. (2008). Loss of parenting self-efficacy among immigrant parents. Contemp. Issues Early Child. 9, 148–160. doi: 10.2304/ciec.2008.9.2.148

[ref2] Alieva A. Hildebrand V. A. Van Kerm P. (2024). The progression of achievement gap between immigrant and native-born students from primary to secondary education. Res. Soc. Stratif. Mobil. 92:100961. doi: 10.1016/j.rssm.2024.100961

[ref3] Aninat I. Vergara R. (2020). Inmigración en Chile: Una Mirada multidimensional. Santiago de Chile: Fondo de Cultura Económica.

[ref4] Antony-Newman M. (2018). Parental involvement of immigrant parents: a meta-synthesis. Educ. Rev. 71, 362–381. doi: 10.1080/00131911.2017.1423278, PMID: 39989647

[ref5] Baker T. L. Wise J. Kelley G. Skiba R. J. (2016). Identifying barriers: creating solutions to improve family engagement. Sch. Community J. 26, 161–184. Available online at: https://www.adi.org/journal/2016fw/BakerEtAlFall2016.pdf (Accessed December, 03, 2024).

[ref6] Ball A. Bates S. Amorose A. Anderson-Butcher D. (2019). The parent perceptions of overall school experiences scale: initial development and validation. J. Psychoeduc. Assess. 37, 251–262. doi: 10.1177/0734282917742310

[ref7] Ballenger J. (2009). “Parental involvement: low socio-economic status (SES) and ethnic minority parents' struggle for recognition and identity” in The struggle for identity in today's schools: cultural recognition in a time of increasing diversity. eds. Jenlink P. M. Townes F. H. (Lanham, Maryland: Rowman & Littlefield Education), 156–168.

[ref8] Bankston C. L. III. (2004). Social capital, cultural values, immigration, and academic achievement: the host country context and contradictory consequences. Sociol. Educ. 77, 176–179. doi: 10.1177/003804070407700205

[ref9] Bardach L. Röhl S. Oczlon S. Schumacher A. Lüftenegger M. Lavelle-Hill R.. (2024). Cultural diversity climate in school: a meta-analytic review of its relationships with intergroup, academic, and socioemotional outcomes. Psychol. Bull. 150, 1397–1439. doi: 10.1037/bul0000454, PMID: 39652446

[ref10] Barger M. M. Kim E. M. Kuncel N. R. Pomerantz E. M. (2019). The relation between parents’ involvement in children’s schooling and children’s adjustment: a meta-analysis. Psychol. Bull. 145, 855–890. doi: 10.1037/bul0000201, PMID: 31305088

[ref11] Bates D. Mächler M. Bolker B. Walker S. (2015). Fitting linear mixed-effects models using lme4. J. Stat. Softw. 67, 1–48. doi: 10.18637/jss.v067.i01

[ref12] Baysu G. Grew E. Hillekens J. Phalet K. (2024). Trajectories of ethnic discrimination and school adjustment of ethnically minoritized adolescents: the role of school diversity climate. Child Dev. 95, 2215–2231. doi: 10.1111/cdev.14133, PMID: 39129254 PMC11579631

[ref13] Beelman A. Heinemann K. S. (2014). Preventing prejudice and improving intergroup attitudes: a meta-analysis of child and adolescent training programs. J. Appl. Dev. Psychol. 35, 10–24. doi: 10.1016/j.appdev.2013.11.002

[ref14] Bennet M. J. (1986). A developmental approach to training for intercultural sensitivity. Int. J. Intercult. Relat. 10, 179–196. doi: 10.1016/0147-1767(86)90005-2

[ref15] Berkowitz R. Astor R. A. Pineda D. DePedro K. T. Weiss E. L. Benbenishty R. (2021). Parental involvement and perceptions of school climate in California. Urban Educ. 56, 393–423. doi: 10.1177/0042085916685764

[ref16] Bhawuk D. Sakuda K. H. Munusamy V. P. “Intercultural competence development and triple-loop cultural learning: toward a theory of intercultural sensivity”. In Ang S. Dyne L. Van (Eds.), Handbook of cultural intelligence: theory, measurements and implications. Armonk, NY.: M.E. Sharpe (2008) p. 342–355.

[ref17] Boonk L. Gijselaers H. J. Ritzen H. Brand-Gruwel S. (2018). A review of the relationship between parental involvement indicators and academic achievement. Educ. Res. Rev. 24, 10–30. doi: 10.1016/j.edurev.2018.02.001

[ref18] Bornstein M. H. (2017). Parenting in acculturation: two contemporary research designs and what they tell us. Curr. Opin. Psychol. 15, 195–200. doi: 10.1016/j.copsyc.2017.03.020, PMID: 28813262 PMC5604855

[ref19] Bornstein M. H. Cote L. R. (2006). “Parenting cognitions and practices in the acculturative process” in Acculturation and parent-child relationships: measurement and development. eds. Bornstein M. H. Cote L. R. (Lawrence Erlbaum Associates Publishers), 173–196.

[ref20] Bourdieu P. Passeron J. C. (1990). Reproduction in education, society and culture, transl. R Nice. Newbury., Park, CA: Sage.

[ref9002] Bronfenbrenner U. Morris P. A. (1998). The ecology of developmental processes. In Damon W. Lerner R. M. (Eds.), Handbook of child psychology: Volume 1: Theoretical models of human development (5th ed.). (pp. 993–1028). Hoboken, NJ: John Wiley & Sons Inc.

[ref21] Byrd C. M. (2017). The complexity of school racial climate: reliability and validity of a new measure for secondary students. Br. J. Educ. Psychol. 87, 700–721. doi: 10.1111/bjep.12179, PMID: 28850714

[ref22] Cabieses B. Oyarte M. (2020). Health access to immigrants: identifying gaps for social protection in health. Rev. Saude Publica 54, 20–13. doi: 10.11606/s1518-8787.2020054001501, PMID: 32074219 PMC7017981

[ref23] Calzada E. J. Huang K. Y. Hernandez M. Soriano E. Acra C. F. Dawson-McClure S.. (2015). Family and teacher characteristics as predictors of parent involvement in education during early childhood among Afro-Caribbean and Latino immigrant families. Urban Educ. 50, 870–896. doi: 10.1177/0042085914534862, PMID: 26417116 PMC4582786

[ref24] Cameron L. Turner R. N. (2017). “Intergroup contact among children” in Intergroup contact theory: recent developments and future directions. eds. Vezzali L. Stathi L. S. (Londres: Routlege), 151–168.

[ref25] Chao M. M. Takeuchi R. Farh J. L. (2017). Enhancing cultural intelligence: the roles of implicit culture beliefs and adjustment. Pers. Psychol. 70, 257–292. doi: 10.1111/peps.12142

[ref9003] Chen G. M. Starosta W. J. (1998). A review of the concept of intercultural awareness. Human Communication, 2, 27–54. 5. Available online at: https://digitalcommons.uri.edu/com_facpubs/37 (Accessed November, 24, 2021).

[ref26] Chen G.-M. Starosta W. J. (2000). The development and validation of the intercultural sensitivity scale. Hum. Commun. 3, 1–15. Available online at: https://digitalcommons.uri.edu/com_facpubs/36 (Accessed November, 24, 2021).

[ref27] Chrobot-Mason D. Aramovich N. P. (2013). The psychological benefits of creating an affirming climate for workplace diversity. Group Org. Manag. 38, 659–689. doi: 10.1177/1059601113509835

[ref28] Davis-Kean P. E. Tighe L. A. Waters N. E. (2021). The role of parent educational attainment in parenting and children’s development. Curr. Dir. Psychol. Sci. 30, 186–192. doi: 10.1177/0963721421993116

[ref29] Domina T. (2005). Leveling the home advantage: assessing the effectiveness of parental involvement in elementary school. Sociol. Educ. 78, 233–249. doi: 10.1177/003804070507800303

[ref30] Eberhard J. P. Lauer C. M. (2019). Diferencias en la elección de establecimiento educacional para la población local e inmigrantes: Caso chileno. Estudios pedagógicos 45, 29–45. doi: 10.4067/S0718-07052019000200029

[ref31] Epstein J. L. (2018). School, family, and community partnerships: preparing educators and improving schools. New York: Routledge.

[ref32] Eyzaguirre S. Aguirre J. Blanco N. (2019). “Dónde estudian, cómo les va y qué impacto tienen los escolares inmigrantes?” in Inmigración en Chile. Una mirada multidimensional. eds. Aninat E. Vergara R. (Santiago de Chile: Fondo de Cultura Económica), 148–186.

[ref33] Fang L. Sun R. C. Yuen M. (2017). Be useful to society: parental academic involvement in rural to urban migrant children‘s education in China. Asia Pac. Educ. Rev. 18, 361–371. doi: 10.1007/s12564-017-9491-8

[ref34] Fine-Davis M. Faas D. (2014). Equality and diversity in the classroom: a comparison of students’ and teachers’ attitudes in six European countries. Soc. Indic. Res. 119, 1319–1334. doi: 10.1007/s11205-013-0547-9

[ref35] Flanagan-Bórquez A. Benavides-Cereceda C. Fuentes-Madariaga C. Kraemer-Ibaceta S. Sepúlveda-Vicencio M. (2021). Estudio sobre las experiencias de docentes chilenos que trabajan con estudiantes inmigrantes en escuelas públicas de la Región de Valparaíso, Chile. Perspectiva Educacional 60, 32–56. doi: 10.4151/07189729-vol.60-iss.3-art.1218

[ref36] Friedman B. A. Bobrowski P. E. Geraci J. (2006). Parents’ school satisfaction: ethnic similarities and differences. J. Educ. Adm. 44, 471–486. doi: 10.1108/09578230610683769

[ref37] Gallardo A. M. (2019). Cogniciones y prácticas parentales en contexto de migración. Summa Psicológica 16, 121–129. doi: 10.18774/0719-448x.2019.16.412

[ref38] García I. (2011). La difícil reproducción de las familias inmigrantes. ¿Hacia la formación de un proletariado étnico español? Papers: Revista de Sociología 96, 55–76. https://raco.cat/index.php/Papers/article/view/228133 (Accessed May, 05, 2013).

[ref39] García L. Y. Friz M. (2019). El efecto migratorio en la asistencia escolar en Chile. Estudios pedagógicos 45, 47–59. doi: 10.4067/S0718-07052019000200047

[ref40] González M. A. (2022). Desigualdad en las trayectorias educativas de jóvenes migrantes: Disquisiciones preliminares. Rev. Actual. Investig. Educ. 22, 1–26. doi: 10.15517/aie.v22i2.48718

[ref41] González J. A. Villarroel A. B. Bastías L. S. (2023). La educación intercultural en Chile. Estrategias pedagógicas en profesores jefes. Palimpsesto 13, 97–116. doi: 10.35588/pa.v13i22.6129, PMID: 39457736

[ref42] González-Falcón I. Arroyo-González M. J. Berzosa-Ramos I. Dusi P. (2022). I do the best I can: the role of immigrant parents in their children’s educational inclusion. Front. Educ. 7:1006026. doi: 10.3389/feduc.2022.1006026

[ref43] Grau M. O. Díaz D. Muñoz Reyes C. (2021). Niñez migrante en chile: Metasíntesis de experiencias educativas con enfoque de derechos. Revista Latinoamericana de Ciencias Sociales, Niñez y Juventud 19, 1–29. doi: 10.11600/rlcsnj.19.2.4228

[ref44] Gubbels J. van der Put C. Assink M. (2019). Risk factors for school absenteeism and dropout: a meta-analytic review. J. Youth Adolesc. 48, 1637–1667. doi: 10.1007/s10964-019-01072-5, PMID: 31312979 PMC6732159

[ref45] Gubbins V. Otero G. (2018). Determinants of parental involvement in primary school: evidence from Chile. Educ. Rev. 72, 137–156. doi: 10.1080/00131911.2018.1487386, PMID: 39989647

[ref46] Gubbins V. Otero G. (2020). Parental involvement and low-SES children’s academic achievement in early elementary school: new evidence from Chile. Educ. Stud. 46, 548–569. doi: 10.1080/03055698.2019.1620691

[ref47] Guthrie C. Andersson H. Cerna L. Borgonovi F. (2019). Strength through diversity: country spotlight report for Chile. OECD education working papers, no. 210. Paris: OECD Publishing.

[ref48] Henrich J. (2020). The WEIRDest people in the world: how the west became psychologically peculiar and particularly prosperous. UK: Penguin.

[ref49] Herrero-Hahn R. Rojas J. G. Montoya-Juárez R. García-Caro M. P. Hueso Montoro C. (2019). Level of cultural self-efficacy of Colombian nursing professionals and related factors. J. Transcult. Nurs. 30, 137–145. doi: 10.1177/1043659618777047, PMID: 29783882

[ref50] Hoover-Dempsey K. V. Sandler H. M. (1995). Parental involvement in children’s education: why does it make a difference? Teach. Coll. Rec. 97, 310–331. doi: 10.1177/016146819509700202

[ref51] Hoover-Dempsey K. V. Sandler H. M. (1997). Why do parents become involved in their children’s education? Rev. Educ. Res. 67, 3–42. doi: 10.3102/00346543067001003

[ref52] Hoover-Dempsey K. V. Walker J. M. Sandler H. M. Whetsel D. Green C. L. Wilkins A. S.. (2005). Why do parents become involved? Research findings and implications. Elem. Sch. J. 106, 105–130. doi: 10.1086/499194

[ref53] Hornby G. Lafaele R. (2011). Barriers to parental involvement in education: an explanatory model. Educ. Rev. 63, 37–52. doi: 10.1080/00131911.2010.488049

[ref54] Instituto Nacional de Estadísticas. (2022). Informe de resultados de la estimación de personas extranjeras residentes en Chile al 31 de diciembre de 2021. Desagregación nacional, regional y principales comunas. Available online at: https://bit.ly/ineysermig (Accessed December, 08, 2024).

[ref55] Ishimaru A. M. Takahashi S. (2017). Disrupting racialized institutional scripts: toward parent–teacher transformative agency for educational justice. Peabody J. Educ. 92, 343–362. doi: 10.1080/0161956X.2017.1324660

[ref56] Joiko S. Vásquez A. (2016). Acceso y elección escolar de familias migrantes en Chile: No tuve problemas porque la escuela es abierta, porque acepta muchas nacionalidades. Calidad en la Educación 2, 132–173. doi: 10.4067/S0718-45652016000200005, PMID: 27315006

[ref57] Jorgensen T. D. Pornprasertmanit S. Schoemann A. M. Rosseel Y. (2022). semTools: Useful tools for structural equation modeling. R package version 0.5-6. Available online at: https://CRAN.R-project.org/package=semTools (Accessed September 13, 2023).

[ref9004] Karakus M. Courtney M. Aydin H. (2023). Understanding the academic achievement of the first-and second-generation immigrant students: A multi-level analysis of PISA 2018 data. Educ. Assess. Eval. Acc. 35, 233–278. doi: 10.1007/s11092-022-09395-x

[ref58] Katıtaş S. Coşkun B. Karadaş H. (2024). The relationship between teachers’ cultural intelligence and multicultural education attitude: the mediating role of intercultural sensitivity. Int. J. Educ. Res. 127:102443. doi: 10.1016/j.ijer.2024.102443

[ref59] Khalfaoui A. García-Carrión R. Villardón-Gallego L. (2020). Bridging the gap: engaging Roma and migrant families in early childhood education through trust-based relationships. Eur. Early Child. Educ. Res. J. 28, 701–711. doi: 10.1080/1350293X.2020.1817241

[ref60] Kim S. (2022). Fifty years of parental involvement and achievement research: a second-order meta-analysis. Educ. Res. Rev. 37:100463. doi: 10.1016/j.edurev.2022.100463

[ref61] Kim S. W. Hill N. E. (2015). Including fathers in the picture: a meta-analysis of parental involvement and students’ academic achievement. J. Educ. Psychol. 107, 919–934. doi: 10.1037/edu0000023

[ref62] Kim I. H. Noh S. (2014). Ethnic and gender differences in the association between discrimination and depressive symptoms among five immigrant groups. J. Immigr. Minor. Health 16, 1167–1175. doi: 10.1007/s10903-013-9969-3, PMID: 24375383

[ref63] Koutsouveli E. Geraki A. (2022). School management and climate to enhance parental involvement. Int. J. Res. Educ. Sci. 8, 662–679. doi: 10.46328/ijres.2921

[ref64] Lahoz I Ubach S. Cordeu Cuccia C. (2021). Sensibilidad intercultural, clima escolar y contacto intergrupal en estudiantes de educación primaria y secundaria de la Región Metropolitana de Santiago de Chile. Revista de Investigación Educativa 39, 131–147. doi: 10.6018/rie.415921

[ref65] Lazović N. Vidosavljević S. Markovic E. Krulj J. (2022). The correlation between father involvement and the academic achievement of their children: meta-analysis. Int. J. Cogn. Res. Sci. Eng. Educ. 10, 53–60. doi: 10.23947/2334-8496-2022-10-3-53-60

[ref66] Lee S. K. Ma E. G. (2019). The multicultural sensitivity and cultural openness of childhood teachers effect on the multicultural teaching efficacy. Educ. Res. Inst. 39, 281–295. doi: 10.34245/jed.39.2.281

[ref67] Li X. Yang H. Wang H. Jia J. (2020). Family socioeconomic status and home-based parental involvement: a mediation analysis of parental attitudes and expectations. Child Youth Serv. Rev. 116:105111. doi: 10.1016/j.childyouth.2020.105111

[ref9005] Lüdecke D. (2020). sjPlot: Data Visualization for Statistics in Social Science. https://cran.r-project.org/package=sjPlot

[ref9006] Marchand A. D. Vassar R. R. Diemer M. A. Rowley S. J. (2019). Integrating race, racism, and critical consciousness in Black parents’ engagement with schools. J. Fam. Theory Rev. 11, 367–384. doi: 10.1111/jftr.12344

[ref9007] Martínez-Taboada C. Mera M. J. Amutio A. Castañeda X. Felt E. Nicolae G. (2017). The Impact of Cultural Dissonance and Acculturation Orientations on Immigrant Students’ academic performance. Revista Universitas Psychologica. 16, 1–14. doi: 10.11144/Javeriana.upsy16-5.cier

[ref68] Martínez-Zelaya G. P. Mera-Lemp M. J. Bilbao M. (2020). Preferencias aculturativas y su relación con la sensibilidad intercultural y el bienestar. Revista de Psicología 29, 88–102. doi: 10.5354/0719-0581.2020.55961

[ref69] McBride B. A. Dyer W. J. Liu Y. Brown G. L. Hong S. (2009). The differential impact of early father and mother involvement on later student achievement. J. Educ. Psychol. 101, 498–508. doi: 10.1037/a0014238, PMID: 25414521 PMC4235963

[ref70] Mendoza Mardones A. I. (2024). Empowering equity: teachers motivations and practices for supporting international migrant students in elementary schools. Urban Rev. 57, 164–198. doi: 10.1007/s11256-024-00718-4

[ref71] Mera-Lemp M. J. Bilbao M. Basabe N. (2020). School satisfaction in immigrant and Chilean students: the role of prejudice and cultural self-efficacy. Front. Psychol. 11:3393. doi: 10.3389/fpsyg.2020.613585, PMID: 33362675 PMC7758508

[ref72] Mera-Lemp M. J. Martínez-Zelaya G. (2021). Relaciones intergrupales en la escuela: cercanía social, prejuicio y aculturación en estudiantes inmigrantes latinoamericanos y chilenos. Inclusão Social 13, 220–233. Available online at: http://hdl.handle.net/20.500.11959/brapci/153226 (Accessed June, 04, 2022).

[ref73] Mera-Lemp M. J. Martínez-Zelaya G. Bilbao M. A. Orellana A. (2021, 2021). “South-south migration in Chile: well-being and intergroup relations between Latin-American immigrants and host society members” in Psychological perspectives on intra-regional migration in Latin America. eds. Smith-Castro V. Sirlopú D. Eller A. Cakal H. (American Psychological Association/APA), 19–37.

[ref74] Mera-Lemp M. J. Torres-Vallejos J. Guglielmetti-Serrano F. (2024). La amenaza cultural y la actitud de docentes chilenos hacia la multiculturalidad en la escuela: el rol de la ansiedad exogrupal y la sensibilidad intercultural. Revista Española de Pedagogía 82, 607–625. doi: 10.22550/2174-0909.4038

[ref75] Ministerio de Desarrollo Social y Familia (2024). Indicadores de integración social de las personas nacidas fuera de Chile. Santiago: Observatorio Social. Ministerio de Desarrollo Social y Familia.

[ref76] Montecinos C. Sisto V. Ahumada L. (2010). The construction of parents and teachers as agents for the improvement of municipal schools in Chile. Comp. Educ. 46, 487–508. doi: 10.1080/03050068.2010.519481

[ref77] Murray B. Domina T. Petts A. Renzulli L. Boylan R. (2020). “We’re in this together”: bridging and bonding social capital in elementary school PTOs. Am. Educ. Res. J. 57, 2210–2244. doi: 10.3102/0002831220908848

[ref78] Mzidabi J. Goudeau S. Delès R. Claes N. Easterbrook M. J. Alexopoulos T.. (2024). Unequal homework: the hidden forces of social class contexts and parental self-efficacy in shaping educational outcomes. J. Soc. Issues 80, 1315–1344. doi: 10.1111/josi.12656

[ref79] Nesteruk O. Marks L. (2011). Parenting in immigration: experiences of mothers and fathers from eastern Europe raising children in the United States. J. Comp. Fam. Stud. 42, 809–825. doi: 10.3138/jcfs.42.6.809

[ref80] Ogbu J. U. Simons H. D. (1998). Voluntary and involuntary minorities: a cultural-ecological theory of school performance with some implications for education. Anthropol. Educ. Q. 29, 155–188. doi: 10.1525/aeq.1998.29.2.155

[ref81] Olmedo I. M. (2003). Accommodation and resistance: Latinas struggle for their children’s education. Anthropol. Educ. Q. 34, 373–395. doi: 10.1525/aeq.2003.34.4.373

[ref82] Onsès J. Segovia S. Sancho J. M. (2023). Migrant families and Children’s inclusion in culturally diverse educational contexts in Spain. Front. Educ. 8:1013071. doi: 10.3389/feduc.2023.1013071

[ref83] Ortega L. Boda Z. Treviño E. Arriagada V. Gelber D. Escribano M. D. R. (2020). The centrality of immigrant students within teacher-student interaction networks: a relational approach to educational inclusion. Teach. Teach. Educ. 95:103126. doi: 10.1016/j.tate.2020.103126

[ref84] Oyarzún J. D. D. Parcerisa L. Carrasco A. (2022). Discriminación étnica y cultural en procesos de elección de escuela en minorías socioculturales en Chile. Psicoperspectivas 21, 87–98. doi: 10.5027/psicoperspectivas-vol21-issue1-fulltext-2537

[ref85] Özdemir M. Bayram Özdemir S. (2020). “Why do some immigrant children and youth do well in school whereas others fail?: current state of knowledge and directions for future research” in Contextualizing immigrant and refugee resilience. eds. Güngör D. Strohmeier D., Advances in immigrant family research (Cham: Springer).

[ref86] Özdemir S. Stattin H. (2013). Why and when is ethnic harassment a risk for immigrant adolescents’ school adjustment? Understanding the processes and conditions. J. Youth Adolesc. 43, 1252–1265. doi: 10.1007/s10964-013-0038-y 24132501

[ref87] Pavez-Soto I. Ortiz-López J. E. Sepúlveda N. Jara P. Olguín C. (2019). Racialización de la niñez migrante haitiana en escuelas de Chile. Interciencia 44, 414–420. Available online at: https://www.redalyc.org/journal/339/33960285007/33960285007.pdf (Accessed January, 16, 2024).

[ref88] Peček M. Čuk I. Lesar I. (2008). Teachers’ perceptions of the inclusion of marginalised groups. Educ. Stud. 34, 225–239. doi: 10.1080/03055690701811347

[ref89] Ram K. Wickham H. (2023). wesanderson: A Wes Anderson Palette Generator. R package version 0.3.7. Available online at: https://github.com/karthik/wesanderson

[ref90] Rane T. R. McBride B. A. (2000). Identity theory as a guide to understanding fathers’ involvement with their children. J. Fam. Issues 21, 347–366. doi: 10.1177/019251300021003004

[ref91] Rania N. Cardinali P. Cifatte C. Migliorini L. (2012). Adolescent adjustment and cultural self-efficacy. Probl. Psychol. 21st Cent. 1, 59–71. Available online at: https://oaji.net/articles/2014/444-1391943053.pdf (Accessed April, 24, 2013).

[ref9008] R Core Team. (2014). R: A language and environment for statistical computing. R Foundation for Statistical Computing.

[ref92] Reynolds A. D. Crea T. M. Medina J. Degnan E. McRoy R. (2015). A mixed methods case study of parent involvement in an urban high school serving minority students. Urban Educ. 50, 750–775. doi: 10.1177/0042085914534272

[ref93] Riedemann A. Stefoni C. Stang F. Corvalán J. (2020). Desde una educación intercultural para pueblos indígenas hacia otra pertinente al contexto migratorio actual. Un análisis basado en el caso de Chile. Estudios atacameños 64, 337–359. doi: 10.22199/issn.0718-1043-2020-0016

[ref94] Rodenborg N. Boisen L. A. (2013). Aversive racism and intergroup contact theories: cultural competence in a segregated world. J. Soc. Work. Educ. 49, 564–579. doi: 10.1080/10437797.2013.812463

[ref95] Rollè L. Gullotta G. Trombetta T. Curti L. Gerino E. Brustia P.. (2019). Father involvement and cognitive development in early and middle childhood: a systematic review. Front. Psychol. 10:2405. doi: 10.3389/fpsyg.2019.02405, PMID: 31708843 PMC6823210

[ref96] Rosseel Y. (2012). lavaan: An R Package for Structural Equation Modeling. J. Stat. Softw. 48, 1–36. doi: 10.18637/jss.v048.i02

[ref9009] RStudioTeam. (2015). RStudio: Integrated Development for R. RStudio, Inc. http://www.rstudio.com/

[ref97] Sandoval R. Echeverría S. Valdés Á. (2017). Participación de los padres en la educación: una prueba del modelo de Hoover-Dempsey y Sandler. Perspect. Educ. 56, 139–153. doi: 10.4151/07189729-Vol.56-Iss.2-Art.495

[ref98] Sari A. C. Yalçinkaya Ö. A. (2023). Mediator role of intergroup anxiety in relationship between the social contact, intercultural sensitivity and attitudes towards Syrians among Turkish local society. Int. Migr. 61, 257–271. doi: 10.1111/imig.13097

[ref99] Schachner M. K. Brenick A. Noack P. Van de Vijver F. J. Heizmann B. (2015). Structural and normative conditions for interethnic friendships in multiethnic classrooms. Int. J. Intercult. Relat. 47, 1–12. doi: 10.1016/j.ijintrel.2015.02.003

[ref100] Schachner M. K. Noack P. Van de Vijver F. J. Eckstein K. (2016). Cultural diversity climate and psychological adjustment at school—equality and inclusion versus cultural pluralism. Child Dev. 87, 1175–1191. doi: 10.1111/cdev.12536, PMID: 27091829

[ref101] Schachner M. K. Schwarzenthal M. Van De Vijver F. J. Noack P. (2019). How all students can belong and achieve: effects of the cultural diversity climate amongst students of immigrant and nonimmigrant background in Germany. J. Educ. Psychol. 111, 703–716. doi: 10.1037/edu0000303

[ref102] Schwarzenthal M. Schachner M. K. Juang L. P. Van De Vijver F. J. (2020). Reaping the benefits of cultural diversity: classroom cultural diversity climate and students’ intercultural competence. Eur. J. Soc. Psychol. 50, 323–346. doi: 10.1002/ejsp.2617

[ref103] Seginer R. (2006). Parents’ educational involvement: a developmental ecology perspective. Parent Sci. Pract. 6, 1–48. doi: 10.1207/s15327922par0601_1

[ref104] Segovia P. Rendón B. (2020). Estudiantes extranjeros/as en la representación de los docentes en una escuela de Santiago: elementos para una educación intercultural. *Polis. Revista Latinoamerican*a 56, 1–22. Available online at: http://journals.openedition.org/polis/19337 (Accessed March, 21, 2024).

[ref105] Segovia-Lagos P. Diaz-Latas A. Roessler-Vergara P. (2023). Acculturation stress and psychological well-being in Latin American immigrant schoolchildren in a district of Santiago de Chile. Sapienza Int. J. Interdiscip. Stud. 4:e23003. doi: 10.51798/sijis.v4i1.576

[ref106] Serna C. Yubero S. Larrañaga E. (2008). Exclusión educativa y social: el contexto social como escenario del fracaso escolar. Bits: Boletín informativo trabajo social 4, 12–23. Available online at: https://www.researchgate.net/profile/Elisa-Larranaga/publication/28264402_Exclusion_educativa_y_social_el_contexto_social_como_escenario_del_fracaso_escolar/links/5848569b08aeda696825e49e/Exclusion-educativa-y-social-el-contexto-social-como-escenario-del-fracaso-escolar.pdf (Accessed July, 10, 2023).

[ref107] Servicio Jesuita a Migrantes. (2021). Una caracterización de la pobreza, el trabajo y la seguridad social en la población migrante (Informe N°1). Santiago, Chile. Available online at: https://www.migracionenchile.cl/publicaciones

[ref108] Servicio Jesuita a Migrantes. (2024) Anuario Estadístico de Movilidad Humana en Chile 2023. Available online at: https://sjmchile.org/wp-content/uploads/2024/06/Anuario-2023.pdf

[ref109] Sheng X. (2012). Cultural capital and gender differences in parental involvement in children's schooling and higher education choice in China. Gend. Educ. 24, 131–146. doi: 10.1080/09540253.2011.582033

[ref9010] Sime D. Fassetta G. McClung M. (2017). ‘It’s good enough that our children are accepted’: Roma mothers’ views of children’s education postmigration, Br. J. Sociol. Educ. doi: 10.1080/01425692.2017.1343125

[ref110] Sirlopú D. Renger D. (2020). Social recognition matters: consequences for school participation and life satisfaction among immigrant students. J. Community Appl. Soc. Psychol. 30, 561–575. doi: 10.1002/casp.2463

[ref111] Sohn S. Wang X. C. (2006). Immigrant parents’ involvement in American schools: perspectives from Korean mothers. Early Childhood Educ. J. 34, 125–132. doi: 10.1007/s10643-006-0070-6

[ref112] Stanley D. J. Spence J. R. (2018). Reproducible tables in psychology using the apaTables package. Adv. Methods Pract. Psychol. Sci. 1, 415–431. doi: 10.1177/2515245918773743

[ref113] Swap S. M. (1993). Developing home-school partnerships: from concepts to practice. New York: Teachers' College Press, Columbia University.

[ref114] Tamam E. Krauss S. E. (2017). Ethnic-related diversity engagement differences in intercultural sensitivity among Malaysian undergraduate students. Int. J. Adolesc. Youth 22, 137–150. doi: 10.1080/02673843.2014.881295

[ref115] Ting-Toomey S. (2009). “Intercultural conflict competence as a facet of intercultural competence development: multiple conceptual approaches” in The Sage handbook of intercultural competence. ed. Deardorff D. (Thousand Oaks, CA: SAGE Publications), 100–120.

[ref116] Titzmann P. F. Brenick A. Silbereisen R. K. (2015). Friendships fighting prejudice: a longitudinal perspective on adolescents’ cross-group friendships with immigrants. J. Youth Adolesc. 44, 1318–1331. doi: 10.1007/s10964-015-0256-6, PMID: 25647141

[ref117] Turney K. Kao G. (2009). Barriers to school involvement: are immigrant parents disadvantaged? J. Educ. Res. 102, 257–271. doi: 10.3200/JOER.102.4.257-271

[ref118] Ulbricht J. Schachner M. K. Civitillo S. Noack P. (2022). Teachers’ acculturation in culturally diverse schools. How is the perceived diversity climate linked to intercultural self-efficacy? Front. Psychol. 13:953068. doi: 10.3389/fpsyg.2022.953068 36337492 PMC9634156

[ref119] Ülger Z. Dette-Hagenmeyer D. E. Reichle B. Gaertner S. L. (2018). Improving outgroup attitudes in schools: a meta-analytic review. J. Sch. Psychol. 67, 88–103. doi: 10.1016/j.jsp.2017.10.002, PMID: 29571537

[ref120] Vandekerckhove A. Aarssen J. (2020). High time to put the invisible children on the agenda: supporting refugee families and children through quality ECEC. Eur. Early Child. Educ. Res. J. 28, 104–114. doi: 10.1080/1350293X.2020.1707366

[ref121] Wilder S. (2023). “Effects of parental involvement on academic achievement: a meta-synthesis” in Mapping the field 75 years of educational review. eds. Martin J. Bowl M. Banks G., vol. II (London: Routledge), 137–157.

[ref122] Yu H. (2020). The making of ‘incompetent parents’: intersectional identity, habitus and Chinese rural migrant‘s parental educational involvement. Aust. Educ. Res. 47, 555–570. doi: 10.1007/s13384-019-00361-z

[ref123] Zhang X. Zhou M. (2019). Interventions to promote learners’ intercultural competence: a meta-analysis. Int. J. Intercult. Relat. 71, 31–47. doi: 10.1016/j.ijintrel.2019.04.006

