# Supplementary material for: Bridging cultures: the role of school’s cultural diversity climate and cultural sensitivity in immigrant parents’ school involvement
Source: Front Psychol. 2025 Mar 27;16:1561863. doi: 10.3389/fpsyg.2025.1561863 (PMC11984563; doi:10.3389/fpsyg.2025.1561863)
Supplement: Supplementary file 2 [file Data_Sheet_2.pdf]

```
# Libraries
library(readxl) #to read data
library(xlsx) #to create an excel archive
library(psych) #to (among others) meta-analysis function
library(GPArotation) #oblimin rotation
library(lavaan) #CFAs,structural models, etc.
library(semTools) #Omegas and reliability
library(tidyverse) #data management, visualization, transformation, etc.
library(RColorBrewer) #colors
library(apaTables) #correlation analyses and regressions
library(ggpubr) #groping figures
library(reshape2) #to change the orientation of data
library(ggridges) # Ridgeline
library(skimr) # Descriptive visualization
library(lme4)
library(sjPlot)
library(sjmisc)
library(wesanderson)

# Set the working directory (syntax and data in the same archive) and
load the data
setwd("C://") # Write the route to the data from your PC
data <- read_excel("data_involvement.xlsx")

colnames(data)
# 0. Pre-processing ####
# 0.1 Sex, Nationality, NEduc, Dependency, Origin

data$Sexo2 <- factor(data$Sexo, levels=c(0,1), labels=c("Men", "Women"))

data$Nac2 <- factor(data$Nac, levels=c(1,2), labels=c("Venezuela",
"Peru"))

data$NEduc2 <- factor(data$NEduc, levels=c(1,2,3,4,5),
                      labels=c("B?sica", "Media", "T?cnico",
"Universitario", "Postgrado"))

data$NEduc2 <- factor(data$NEduc, levels=c(1,2,3,4,5),
                      labels=c("Elementary", "High-school", "Technical",
"University", "Post-graduate"))

data$Dependencia2 <- factor(data$Dependencia, levels=c(1,2),
labels=c("Public", "Charter"))

data$Curso2 <- factor(data$Curso, levels=c(1,2,3,4),
                      labels=c("First (6-7 y/o)", "Second (7-8 y/o)",
"Third (8-9 y/o)", "Fourth (9-10 y/o)"))

data %>%
  group_by(Dependencia) %>%
  count()

# 0.2 Item recoding ####
```

```
# Recoding the inversed items
rescale1_5 <- function(x_i){
  6-x_i
}

rescale1_4 <- function(x_i){
  5-x_i
}

data$SI2r <- sapply(data$SI2, rescale1_5)
data$SI4r <- sapply(data$SI4, rescale1_5)
data$SI9r <- sapply(data$SI9, rescale1_5)
data$SI12r <- sapply(data$SI12, rescale1_5)
data$SI15r <- sapply(data$SI15, rescale1_5)
data$SI18r <- sapply(data$SI18, rescale1_5)
data$SI20r <- sapply(data$SI20, rescale1_5)
data$SI22r <- sapply(data$SI22, rescale1_5)

# check the transformations
data %>%
  select(SI2, SI2r, SI4, SI4r) %>%
  cor(use="complete.obs")

data$C1_r <- sapply(data$C1_, rescale1_4)
data$C3_r <- sapply(data$C3_, rescale1_4)
data$C4_r <- sapply(data$C4_, rescale1_4)
data$C6_r <- sapply(data$C6_, rescale1_4)
data$C7_r <- sapply(data$C7_, rescale1_4)
data$C8_r <- sapply(data$C8_, rescale1_4)
data$C10_r <- sapply(data$C10_, rescale1_4)
data$C14_r <- sapply(data$C14_, rescale1_4)
data$C16_r <- sapply(data$C16_, rescale1_4)

# check the transformations
data %>%
  select(C1_, C1_r, C7_, C7_r) %>%
  cor(use="complete.obs")


# 1. Reliability analyses and Scale construction ####
colnames(data)

# 1.1. Cultural Diversity Climate
# Affirming Climate for Workplace Diversity (Chrobot-Mason & Aramovich,
2013)

checkgroup_alpha_climap <- data %>%
  select(C2, C5, C9, C11, C12, C13, C15, C17, C18) %>%
  do(alpha = psych::alpha(.))

checkgroup_alpha_climan <- data %>%
  select(C1_r, C3_r, C4_r, C6_r, C7_r, C8_r, C10_r, C14_r, C16_r) %>%
  do(alpha = psych::alpha(.))
```

```
checkgroup_alpha_climatot <- data %>%
  select(C1_r, C2,  C3_r, C4_r, C5, C6_r, C7_r, C8_r, C9, C10_r, C11,
C12, C13, C14_r, C15, C16_r, C17, C18) %>%
  do(alpha = psych::alpha(.))

checkgroup_alpha_climap$alpha[1]
checkgroup_alpha_climan$alpha[1]
checkgroup_alpha_climatot$alpha[1] # alpha = .93


# 1.2. Cultural Sensitivity:
# The cultural sensitivity scale (Chen & Starosta, 2000) validated in
Chile by Mart?nez-Zelaya et al. (2020)

checkgroup_alpha_culs <- data %>%
  select(SI1, SI2r, SI3, SI4r, SI5, SI6, SI7, SI8, SI9r, SI10,
         SI11, SI12r, SI13, SI14, SI15r, SI16, SI17, SI18r, SI19, SI20r,
         SI21, SI22r, SI23, SI24) %>%
  do(alpha = psych::alpha(.))

checkgroup_alpha_culs$alpha[1] # alpha = .91


# 1.3. Parental Involvement
# School Involvement Questionnaire of the Metropolitan Survey on Family
and Education

checkgroup_alpha_home <- data %>%
  select(IEH1, IEH2, IEH3, IEH4) %>%
  do(alpha = psych::alpha(.))

checkgroup_alpha_school <- data %>%
  select(IEF1, IEF2, IEF3, IEF4, IEF5, IEF6, IEF7) %>%
  do(alpha = psych::alpha(.))

checkgroup_alpha_total <- data %>%
  select(IEH1, IEH2, IEH3, IEH4, IEF1, IEF2, IEF3, IEF4, IEF5, IEF6,
IEF7) %>%
  do(alpha = psych::alpha(.))

checkgroup_alpha_home$alpha[1] # alpha = .90
checkgroup_alpha_school$alpha[1] # alpha = .87
checkgroup_alpha_total$alpha[1] # alpha = .88


# 1.2 Variable creation ####
data <- data %>%
  mutate(
    # Independent Variables
    Clima2=rowMeans(data[,c("C1_r", "C2",  "C3_r", "C4_r", "C5", "C6_r",
"C7_r", "C8_r", "C9", "C10_r", "C11", "C12", "C13", "C14_r", "C15",
"C16_r", "C17", "C18")], na.rm=TRUE),
    Sens_cul= rowMeans(data[,c("SI1", "SI2r", "SI3", "SI4r", "SI5",
"SI6", "SI7", "SI8", "SI9r", "SI10",
```

```
                              "SI11", "SI12r", "SI13", "SI14", "SI15r",
"SI16", "SI17", "SI18r", "SI19", "SI20r",
                              "SI21", "SI22r", "SI23", "SI24")],
na.rm=TRUE),

    # Dependent variable
    Inv_h=rowMeans(data[,c("IEH1", "IEH2", "IEH3", "IEH4")], na.rm=TRUE),
#hogar
    Inv_e=rowMeans(data[,c("IEF1", "IEF2", "IEF3", "IEF4", "IEF5",
"IEF6", "IEF7")], na.rm=TRUE), # escuela
    Inv_tot=rowMeans(data[,c("IEH1", "IEH2", "IEH3", "IEH4", "IEF1",
"IEF2", "IEF3", "IEF4", "IEF5", "IEF6", "IEF7")], na.rm=TRUE)
  )


# 1.3. Correlation analyses
colnames(data)

data %>%
  filter(!is.na(Dependencia)) %>%
  select(Sexo, Edad, Nac, NEduc, Estancia, Dependencia, Tiempo_apod,
Curso, Estancia,  Clima2, Sens_cul, Inv_h, Inv_e, Inv_tot) %>%
  apa.cor.table(filename = "correlaciones_all.doc")

data %>%
  filter(!is.na(Dependencia)) %>%
  select(Sexo, Edad, Nac, NEduc, Estancia, Dependencia, Tiempo_apod,
Curso, Estancia,  Clima2, Sens_cul, Inv_h, Inv_e, Inv_tot) %>%
  skimr::skim()

# 1.4 Descriptive ####
colnames(data)

gender <- data %>%
  filter(!is.na(Sexo2)) %>%
  group_by(Sexo2) %>%
  summarise(n = n(), prop = n()/nrow(data)) %>%
  mutate(Type = "Gender")

sex_plot <- ggplot(gender, aes(x = Type, y =prop, fill = Sexo2)) +
  geom_col() + #scale_fill_manual(values = wes_palette("Zissou1")) +
  scale_fill_manual(values = c("#5DAABC","#E86F00")) +
  labs(subtitle = "          Sex", y = NULL, x =NULL) +
  theme_pubclean() +
  #theme_minimal(base_size = 14) +
  coord_flip() +
  guides(fill = guide_legend(reverse=T)) +
  theme(legend.position="bottom") +
  labs(fill = NULL) +
  #ggtitle("Gender") +
  theme(axis.title.y=element_blank(),
        axis.text.y=element_blank(),
        axis.ticks.y=element_blank())
```

```
nationality <- data %>%
  filter(!is.na(Nac2)) %>%
  group_by(Nac2) %>%
  summarise(n = n(), prop = n()/nrow(data)) %>%
  mutate(Type = "Nationality")

nat_plot <- ggplot(nationality, aes(x = Type, y =prop, fill = Nac2)) +
  geom_col() + #scale_fill_manual(values = wes_palette("Zissou1")) +
  scale_fill_manual(values = c("#5DAABC","#E86F00")) +
  labs(subtitle = "          Country of origin", y = NULL, x =NULL) +
  theme_pubclean() +
  #theme_minimal(base_size = 14) +
  coord_flip() +
  guides(fill = guide_legend(reverse=T)) +
  theme(legend.position="bottom") +
  labs(fill = NULL) +
  #ggtitle("Gender") +
  theme(axis.title.y=element_blank(),
        axis.text.y=element_blank(),
        axis.ticks.y=element_blank())

age_plot <- data %>%
  ggplot(aes(x = Edad, fill = Sexo2)) +
  #geom_density(alpha=.5) +
  geom_density(alpha=.8, color = NA) +
  #scale_fill_manual(values = wes_palette("Zissou1")) +
  #scale_fill_gradient(values = wes_palette("Zissou1", 8, type =
"continuous")) +
  scale_fill_manual(values = c("#5DAABC","#E86F00")) +
  theme_pubclean()+
  labs(subtitle = "Proportion by sex", x = "Age in years", y = "Density",
fill = NULL) +
  #theme(axis.title.x = element_text(size = 10)) +
  theme(legend.position="none",
        axis.title.x = element_text(size = 10),
        axis.title.y = element_text(size = 10))

education <- data %>%
  filter(!is.na(NEduc2)) %>%
  group_by(NEduc2) %>%
  summarise(n = n(), prop = n()/nrow(data)) %>%
  mutate(Type = "Education")

edu_plot <- ggplot(education, aes(x = Type, y =prop, fill = NEduc2)) +
  geom_col() +
  #geom_col(position="dodge") +
  scale_fill_manual(values = wes_palette("Zissou1", 8, type =
"continuous")) +
  labs(subtitle = "        Educational level", y = NULL, x =NULL) +
  theme_pubclean() + scale_y_reverse() +
  #theme_minimal(base_size = 14) +
  coord_flip() +
  guides(fill = guide_legend(ncol=5, nrow=1)) +
  theme(legend.position="bottom") +
```

```
  labs(fill = NULL) +
  theme(axis.title.y=element_blank(),
        axis.text.y=element_blank(),
        axis.ticks.y=element_blank())

year <- data %>%
  filter(!is.na(Curso)) %>%
  group_by(Curso2) %>%
  summarise(n = n(), prop = n()/nrow(data)) %>%
  mutate(Type = "Curso")

year_plot <- year %>%
  ggplot(aes(x = Curso2, y = n, fill = Curso2)) +
  geom_bar(stat="identity")+
  labs(subtitle = "Studies Identified", x = "Year", y = "Frequency (n)")+
  theme_pubclean() + rremove("legend") +
  scale_fill_manual(values = wes_palette("Zissou1", 4, type =
"continuous")) +
  labs(subtitle = "        Academic year", y = NULL, x =NULL) +
  theme_pubclean() + rremove("legend") +
  coord_flip()


descriptivos <- ggarrange(ggarrange(sex_plot, nat_plot, year_plot,
ncol=3, labels = c("A", "B", "C")),
          edu_plot, ncol = 1, nrow = 2,
          labels = c("", "D"))

png("1_Descriptive.png", width = 6500, height = 3800, res=1000)
descriptivos
dev.off()


# Ridges plot - Involvement
# Data preparation
data %>%
  select(c(16:26, 2)) %>% # only involvement
  skimr::skim()

data_ridges <- data %>%
  select(c(16:26, 2))

describe(data_ridges)
colnames(data_ridges)

# change the order of the data (from columns to rows)
data_ridges2 <- data_ridges %>%
  gather(key = Activity, value = "Frequency", -Sexo)

data_ridges2 <- data_ridges2 %>%
  group_by(Activity) %>%
  mutate(ID = row_number()) #this creates numbers that re-initiate

data_ridges2 %>%
```

```
  group_by(Activity) %>%
  count()

data_ridges2$Activity2 <-
  factor(data_ridges2$Activity,
         levels=c("IEH1", "IEH2", "IEH3", "IEH4", "IEF1", "IEF2", "IEF3",
"IEF4", "IEF5", "IEF6", "IEF7"),
         labels=c("1. Studying with him/her",
                  "2. Teaching study strategies",
                  "3. Analyzing difficulties",
                  "4. Resolving doubts",
                  "5. Attending ceremonies",
                  "6. Attending parent meetings",
                  "7. Attending meetings with teachers",
                  "8. Attending sports activities",
                  "9. Attending academic activities",
                  "10. Attending cultural activities",
                  "11. Attending activities for parents"
                  ))

data_ridges2$Sex <-
  factor(data_ridges2$Sexo,
         levels=c(0,1),
         labels=c("Father",
                  "Mother"
         ))


part_ridges = data_ridges2 %>%
  filter(!is.na(Frequency)) %>%
  ggplot(aes(x = Frequency, y = fct_rev(as_factor(Activity2)), fill =
Activity2)) +
  geom_density_ridges(alpha = .8) +
  #scale_color_viridis_d() +
  #scale_fill_viridis_d() +
  theme_pubclean() +
  scale_color_manual(values = wes_palette("Zissou1", 11, type =
"continuous")) +
  scale_fill_manual(values = wes_palette("Zissou1", 11, type =
"continuous")) +
  labs(subtitle="", x = NULL, y = NULL, fill = NULL) +
  theme_minimal(base_size = 14) +
  theme(legend.position="none") +
  facet_wrap(~Sex)+
  labs(subtitle = "Involvement")+
  scale_x_discrete(limits = c(1,2,3,4,5), #this last is to add values to
numeric axis
                   labels = c("never", "2", "3", "4", "always"),
                   guide = guide_axis(angle = 25)) +
  theme(axis.text.x= element_text(size=9),
        axis.text.y = element_text(size=9))

png("1_Ridges.png", width = 6000, height = 3300, res=650)
part_ridges
```

```
dev.off()

# Figure altogether
descriptivos2 <- ggarrange(ggarrange(ggarrange(ggarrange(sex_plot,
nat_plot, ncol=1, labels = c("A", "D")),
          year_plot, ncol = 2, labels = c("", "B")), edu_plot, ncol = 1,
labels = c("", "E")),
          part_ridges, ncol = 2, labels = c("", "C"))

png("1_Descriptive_all.png", width = 11000, height = 5500, res=1000)
descriptivos2
dev.off()



# 2. Graphic Models: Relationship between Variables ####
# 2.1. Climate and Involvement

# This section includes a visual assesment of the variables with the goal
# of having a better understanding of their underlying structure.
# This information will be used to make a better selection of the
statistical
# tests used to test the main hypotheses.

clima_invo <- data %>% ggplot()+
  geom_point(aes(x = Clima2, y = Inv_tot, fill  = Centro2, group =
Centro2),
             size=1, alpha=.5,position = "jitter", shape=21,
col="white")+
  geom_smooth(aes(x = Clima2, y = Inv_tot, colour = Centro2, group =
Centro2),
              method = lm, alpha=.5, se=F)+ theme_pubclean() +
  theme(legend.position = "none")+
  scale_color_manual(values = wes_palette("FantasticFox1", 101, type =
"continuous"))+ # Regressions
  scale_fill_manual(values = wes_palette("FantasticFox1", 171, type =
"continuous"))+ # Dots
  labs(title = "Cultural Diversity Climate and Involvement at School",
       #subtitle = " Linear relations between variables across 171
schools",
       x = "Cultural diversity climate", y = "Involvement at school")

png("2_Climate_involve.png", width = 3500, height = 2500, res=650)
clima_invo
dev.off()

# 2.2. Sensitivity and Involvement
sens_invo <- data %>% ggplot()+
  geom_point(aes(x = Sens_cul, y = Inv_tot, fill  = Centro2, group =
Centro2),
             size=1, alpha=.5,position = "jitter", shape=21,
col="white")+
  geom_smooth(aes(x = Sens_cul, y = Inv_tot, colour = Centro2, group =
Centro2),
```

```
             method = lm, alpha=.5, se=F)+  theme_pubclean() +
  theme(legend.position = "none")+
  scale_color_manual(values = wes_palette("FantasticFox1", 101, type =
"continuous"))+ # Regressions
  scale_fill_manual(values = wes_palette("FantasticFox1", 171, type =
"continuous"))+ # Dots
  labs(title = "Cultural Sensitivity and Involvement at School",
       #subtitle = " Linear relations between variables across 171
schools",
       x = "Cultural sensitivity", y = "Involvement at school")

png("2_Sens_involve.png", width = 3500, height = 2500, res=650)
sens_invo
dev.off()

relaciones <- ggarrange(clima_invo, sens_invo, ncol = 1, nrow = 2,
                        labels = c("A", "B"))

png("2 Clima_sens.png", width = 6500, height = 8000, res=1000)
relaciones
dev.off()



# 3. Multilevel Models ####
# Due to the true nature of the relationship among the variables (i.e.,
nested in
# the schools), we conducted the main regressions using multilevel
modeling.
# First, we change the names of the variables so that they appear with
more clarity
# in the figures.

colnames(data)
data_mod <- data
names(data_mod)[names(data_mod) == "Sexo"] <- "Sex"
names(data_mod)[names(data_mod) == "Edad"] <- "Age"
names(data_mod)[names(data_mod) == "Nac"] <- "Nationality"
names(data_mod)[names(data_mod) == "NEduc"] <- "Edu._level"
names(data_mod)[names(data_mod) == "Estancia"] <- "Stay"
names(data_mod)[names(data_mod) == "Dependencia"] <- "Dependency"
names(data_mod)[names(data_mod) == "Tiempo_apod"] <- "Time_guardian"
names(data_mod)[names(data_mod) == "Curso"] <- "Ac._year"
names(data_mod)[names(data_mod) == "Sens_cul"] <- "CS"
names(data_mod)[names(data_mod) == "Clima2"] <- "CDC"
names(data_mod)[names(data_mod) == "InmxCom"] <- "Immigrants"
colnames(data_mod)

# 3.1. Prediction of parental involvement ####
# Total involvement
mod_inv_tot <- lmer(Inv_tot ~ Sex + Age + Nationality + Edu._level + Stay
+
                      Dependency + Time_guardian + Ac._year +
                      CDC + CS + Immigrants +
```

```
                    (1+Nationality|Centro), data=data_mod, na.action =
na.omit,
                  control = lmerControl("Nelder_Mead"))
tab_model(mod_inv_tot, show.df=TRUE, show.std = TRUE, show.fstat = TRUE,
auto.label = TRUE)

# Involvement at home
mod_inv_h <- lmer(Inv_h ~ Sex + Age + Nationality + Edu._level + Stay +
                    Dependency + Time_guardian + Ac._year +
                    CDC + CS + Immigrants +
                    (1+Nationality|Centro), data=data_mod, na.action =
na.omit,
                  control = lmerControl("Nelder_Mead"))
tab_model(mod_inv_h, show.df=TRUE, show.std = TRUE, show.fstat = TRUE,
auto.label = TRUE)

# Involvement at school
mod_inv_e <- lmer(Inv_e ~ Sex + Age + Nationality + Edu._level + Stay +
                    Dependency + Time_guardian + Ac._year +
                    CDC + CS + Immigrants +
                    (1+Nationality|Centro), data=data_mod, na.action =
na.omit,
                  control = lmerControl("Nelder_Mead"))
tab_model(mod_inv_e, show.df=TRUE, show.std = TRUE, show.fstat = TRUE,
auto.label = TRUE)

# 3.2. Visual depiction of the models ####
colors_pos_neg = c("#6FB2C1", "#EF5703")

invo_tot_mod <-
  plot_model(mod_inv_tot, type = "std", colors = colors_pos_neg) +
  labs(title = "Total score", #subtitle = "Predition of involvement at
school (total)",
       y = "Standardized coefficients") +
  ylim(-.2, .3) + geom_hline(yintercept = 0, linetype="dashed", size =
0.9, color = "grey")+
  theme_pubclean()

invo_h_mod <-
  plot_model(mod_inv_h, type = "std", colors = colors_pos_neg) +
  labs(title = "Home activities", #subtitle = "Predition of involvement
at school (home)",
       y = "Standardized coefficients") +
  ylim(-.2, .3) + geom_hline(yintercept = 0, linetype="dashed", size =
0.9, color = "grey")+
  theme_pubclean()

invo_e_mod <-
  plot_model(mod_inv_e, type = "std", colors = colors_pos_neg) +
  labs(title = "School activities", #subtitle = "Predition of involvement
at school (school)",
       y = "Standardized coefficients") +
  ylim(-.2, .3) + geom_hline(yintercept = 0, linetype="dashed", size =
0.9, color = "grey")+
```

```
  theme_pubclean()


models <- ggarrange(invo_tot_mod, ggarrange(invo_h_mod, invo_e_mod,
nrow=2,
                                             labels = c("B", "C")),
                        ncol = 2, nrow = 1,
                        labels = c("A"))

png("3_Multilevel_models.png", width = 6500, height = 3800, res=720)
models
dev.off()

# Nationality
nat_cdc = plot_model(mod_inv_tot, type = "eff", terms = c("CDC",
"Nationality")) +
  labs(title = "", #subtitle = "Predition of involvement at school
(total)",
       y = NULL, x = "Cultural Diversity Climate") + theme_pubclean() +
  scale_color_manual(values = c("#6FB2C1", "#EF5703"),
                     labels = c("Venezuela", "Peru"))+
  scale_fill_manual(values = c("#6FB2C1", "#EF5703"))

nat_cs = plot_model(mod_inv_tot, type = "eff", terms = c("CS",
"Nationality")) +
  labs(title = "", #subtitle = "Predition of involvement at school
(total)",
       y = NULL, x = "Cultural Sensitivity") + theme_pubclean() +
  scale_color_manual(values = c("#6FB2C1", "#EF5703"),
                     labels = c("Venezuela", "Peru"))+
  scale_fill_manual(values = c("#6FB2C1", "#EF5703"))

# Segmented by educational level
el_cdc = plot_model(mod_inv_tot, type = "eff", terms = c("CDC",
"Edu._level")) +
  labs(title = "", #subtitle = "Predition of involvement at school
(total)",
       y = NULL, x = "Cultural Diversity Climate") + theme_pubclean() +
  scale_color_manual(values = c("#88BAAE", "#CAC656", "#E8C31E",
"#E2B306", "#E86F00"),
                     labels=c("Elementary", "High-school", "Technical",
"University", "Post-graduate"))+
  scale_fill_manual(values = c("#88BAAE", "#CAC656", "#E8C31E",
"#E2B306", "#E86F00"))

el_cs = plot_model(mod_inv_tot, type = "eff", terms = c("CS",
"Edu._level")) +
  labs(title = "", #subtitle = "Predition of involvement at school
(total)",
       y = NULL, x = "Cultural Sensitivity") + theme_pubclean() +
  scale_color_manual(values = c("#88BAAE", "#CAC656", "#E8C31E",
"#E2B306", "#E86F00"),
                     labels=c("Elementary", "High-school", "Technical",
"University", "Post-graduate"))+
```

```
  scale_fill_manual(values = c("#88BAAE", "#CAC656", "#E8C31E",
"#E2B306", "#E86F00"))

# Segmented by school dependency
dep_cdc = plot_model(mod_inv_tot, type = "eff", terms = c("CDC",
"Dependency")) +
  labs(title = "", #subtitle = "Predition of involvement at school
(total)",
       y = NULL, x = "Cultural Diversity Climate") + theme_pubclean() +
  scale_color_manual(values = c("#6FB2C1", "#EF5703"),
                     labels = c("Public", "Charter"))+
  scale_fill_manual(values = c("#6FB2C1", "#EF5703"))

dep_cs = plot_model(mod_inv_tot, type = "eff", terms = c("CS",
"Dependency")) +
  labs(title = "", #subtitle = "Predition of involvement at school
(total)",
       y = NULL, x = "Cultural Sensitivity") + theme_pubclean() +
  scale_color_manual(values = c("#6FB2C1", "#EF5703"),
                     labels = c("Public", "Charter"))+
  scale_fill_manual(values = c("#6FB2C1", "#EF5703"))



extra = ggarrange(ggarrange(nat_cdc, nat_cs, common.legend = T, legend =
c("bottom"), nrow = 1, labels = c("A", "")),
          ggarrange(el_cdc, el_cs, common.legend = T,legend =
c("bottom"), nrow = 1, labels = c("B", "")),
          ggarrange(dep_cdc, dep_cs, common.legend = T, legend =
c("bottom"), nrow = 1, labels = c("C", "")),
          nrow =3)

png("3_Coefficients_by_levels.png", width = 7000, height = 8000,
res=1000)
extra
dev.off()



# 4. Mediation Model ####
# Finally, we conducted the mediation model of indirect effects through
path
# analyses

# School involvement predicted by (first) diversity climate and (then)
cultural sensitivity
mod_invol_sep <- '
Sens_cul ~ a*Clima2

Inv_h ~ c1*Clima2 + b1*Sens_cul
Inv_e ~ c2*Clima2 + b2*Sens_cul

Indirecto_h := a * b1
Indirecto_e := a * b2
```

```
total_effect_h := c1 + (a * b1)
total_effect_e := c1 + (a * b2)
'
fit_invol_sep <- sem(mod_invol_sep, data=data, estimator="ML", std.lv =
TRUE)
summary(fit_invol_sep, fit.measures=TRUE)
fit_invol_sep <- parameterestimates(fit_invol_sep, boot.ci.type =
"bca.simple", standardized = TRUE, rsquare = TRUE, output = "data.frame")
write.xlsx(fit_invol_sep, file="Mediations.xlsx", sheetName = "first",
append = TRUE)


# The end ;)
```
